# Aged microglia promote peripheral T cell infiltration by reprogramming the microenvironment of neurogenic niches

**DOI:** 10.1186/s12979-022-00289-6

**Published:** 2022-07-25

**Authors:** Xiaotao Zhang, Rui Wang, Haoran Chen, Chenghao Jin, Ziyang Jin, Jianan Lu, Liang Xu, Yunrong Lu, Jianmin Zhang, Ligen Shi

**Affiliations:** 1 Department of Neurosurgery, Second Affiliated Hospital, School of Medicine, Zhejiang University, 88 Jiefang Road, Hangzhou, 310009 Zhejiang China; 2 Clinical Research Center for Neurological Diseases of Zhejiang Province, Hangzhou, China; 3 Department of Psychiatry, Second Affiliated Hospital, School of Medicine, Zhejiang University, Hangzhou, Zhejiang China; 4 Brain Research Institute, Zhejiang University, Hangzhou, Zhejiang China; 5 Collaborative Innovation Center for Brain Science, Zhejiang University, Hangzhou, Zhejiang China

**Keywords:** Aging, Microglia, Endothelial cells, T cells, Blood–brain barrier, Single-cell transcriptome

## Abstract

**Background:**

The immune cell compartment of the mammalian brain changes dramatically and peripheral T cells infiltrate the brain parenchyma during normal aging. However, the mechanisms underlying age-related T cell infiltration in the central nervous system remain unclear.

**Results:**

Chronic inflammation and peripheral T cell infiltration were observed in the subventricular zone of aged mice. Cell-cell interaction analysis revealed that aged microglia released CCL3 to recruit peripheral CD8^+^ memory T cells. Moreover, the aged microglia shifted towards a pro-inflammation state and released TNF-α to upregulate the expression of VCAM1 and ICAM1 in brain venous endothelial cells, which promoted the transendothelial migration of peripheral T cells. In vitro experiment reveals that human microglia would also transit to a chemotactic phenotype when treated with CSF from the elderly.

**Conclusions:**

Our research demonstrated that microglia play an important role in the aging process of brain by shifting towards a pro-inflammation and chemotactic state. Aged microglia promote T cell infiltration by releasing chemokines and upregulating adhesion molecules on venous brain endothelial cells.

**Supplementary Information:**

The online version contains supplementary material available at 10.1186/s12979-022-00289-6.

## Background

Brain aging is accompanied by T cell accumulation [1–3]. The infiltrated T cells and their secreted cytokines lead to a loss of functional neural stem cells in the subventricular zone (SVZ) [4, 5]. They also attenuate neurogenesis and neuroplasticity of aging brain, manifested by progressive cognitive decline [6, 7]. However, the mechanisms underlying T cell infiltration in the aging SVZ remain elusive, which is fundamentally important for preventing brain aging.

The blood–brain barrier (BBB), which is composed of and regulated by endothelial cells, the basement membrane, pericytes, the glia limitans, and microglia, serves as a relay station between the circulation and the central nervous system (CNS) [8]. Leukocyte migration across the BBB is a complex process, which is triggered by inflammation and chemotactic signals released from the CNS [9, 10]. Once the signals are received, the T cells that express corresponding receptors are arrested by brain endothelial cells (BECs) and then cross the BBB [11, 12]. Age-dependent changes in this process have partially been documented. BBB breakdown has been proven as a consistent feature in aging humans and rodents [13] and manifests as a loss of tight junction integrity and altered transport properties [14–16]. Accumulating evidence supports that the aging BECs show a zonation-dependent, rather than a consistent, change across the vascular bed [14]. However, it is still unclear which part of the aging BECs allow peripheral immune cells to infiltrate the brain. Besides, the mechanisms underlying the BEC changes in the aging brain remain elusive. Our previous study indicated that a unique type of highly-activated microglia could evoke brain inflammation in aged mice [17]. We therefore hypothesized that these senescent microglia were responsible for T cells accumulation, and the inflammatory factors they released might lead to BECs activation, thereby promoting T cells infiltration.

In the present study, we established interaction networks among BECs, microglia, and T cells by analyzing single-cell transcriptional profiles of cells from aged and young mice. We identified a chronic inflammation phenotype with T cell infiltration in the aged SVZ. The circulating T cells were recruited by aged microglia and entered the brain though anchoring adhesion molecules (VCAM1 and ICAM1) on venous BECs. Our findings provide a possible cause for age-related brain inflammation and may help identify potential therapeutic targets.

## Materials and methods

### Animals

Young (8–10 weeks old) C57BL/6 males and females were obtained from SLAC Laboratory Animal Company Limited (Shanghai, China). 20 aged (18 months old) male C57BL/6 mice and 10 aged female (18 months old) were purchased from Beijing Vital River Laboratory Animal Technology Co. Ltd. (Beijing, China). The mice were housed in plastic cages with controlled temperature and humidity and a 12/12-h light/dark cycle. All animal experiment protocols were approved by the Institutional Ethics Committee of the Second Affiliated Hospital, Zhejiang University School of Medicine and were in compliance with the Guide for the Care and Use of Laboratory Animals of the National Institutes of Health. For microglia depletion, PLX5622 was supplied to mice in the diet (Research Diets) at 1200 PPM (1200 mg/kg of chow) for consecutive 7 days.

### Immunostaining of brain sections and image analysis

Mice were deeply anesthetized and perfused transcardially with 25 mL of ice-cold phosphate-buffered saline (PBS), followed by 20 mL of 4% paraformaldehyde solution in PBS. Brains were postfixed in 4% paraformaldehyde for 24 h and dehydrated in serial 15 and 30% sucrose solutions at 4 °C. Then the brain samples were sectioned into coronal slices (25 μm thick). Brain sections were stored in cryoprotectant (40% PBS, 30% glycerol, 30% ethylene glycol) and kept at − 20 °C until immunostaining. Sections were washed twice with PBS, followed by permeabilization in 0.5% Triton X-100 at room temperature. Then sections were blocked with 5% normal donkey serum in PBS for 1 h at room temperature and incubated overnight at 4 °C with the following primary antibodies: anti-CD31 (Santa Cruz, sc18916, 1:50), anti-CD8a (Abcam, ab217344, 1:250), anti-IBA1 (Abcam, ab5076, 1:250), anti-CCL3 (Abcam, ab179638, 1:200), anti-CCL4 (Abcam, ab45690, 1:200), anti-mouse IgG-Alexa Fluor 488 (CST, 4408S, 1:200), anti-VCAM1 (Abcam, ab134047, 1:200), anti-ICAM1 (Abcam, ab109361, 1:100), and anti-TNF-α (Abcam, ab183218, 1:100). Then, the sections were incubated in the dark with donkey secondary antibody conjugated with Alexa Fluor 488, 555, or 594 (Invitrogen, 1:500) at room temperature for 1 h. After washing with PBS three times, the sections were mounted on glass slides with mount-G containing DAPI (Yeasen Biotech). Sections were observed with a Leica TCS SP8 confocal microscope (Leica Microsystems).

Images were adjusted for brightness and contrast using Fiji 2.1.0/1.53c. All confocal images were represented as maximum intensity projections. For cells quantification, three to five randomly selected microscopic regions were captured in each section, then the images were loaded into imageJ and positively stained cells were electronically labeled with the software to avoid duplicated counting. For quantification of IgG extravasation, the parenchymal staining of IgG was slected based on the pre-set threshold petameters. The integrated density of IgG fluorescence of the randomly chosen regions with same size were recorded.

### Flow cytometry

Spleen cells were prepared as described previously [18]. Briefly, the spleen was mechanically dissociated and passed through a 70um filter. The final 4 ml suspension was layered onto 2 ml Ficoll-Paque (GE,17–1440-02) and the cells at the interface were collected after certification (500 g,20 min,4 °C). Single cell samples were incubated with antibodies to surface antigens for 30 minutes on ice at 4 °C in the dark. Fluorochrome compensation was performed with single-stained UltraComp eBeads. Flow cytometery was performed on the BD LSRFortessa flow cytometer (BD biosciences). Data analysis were performed using Flowjo software.

The antibodies used for profiling splenic T cells included anti-mouse CD45-Pacific Blue (1:200; BioLegend, 103,126); anti-mouse CD3e-FITC (1:200; BD Pharmingen, 553,062); anti-mouse CD4-APC-Cy7 (1:200; BD Pharmingen, 552,051); anti-mouse CD8-Percp (1:200; BioLegend, 100,732); anti-mouse CD44-V500 (1:200; BD Pharmingen, 560,781); anti-mouse CD62L-BUV395 (1:200; BD Pharmingen, 740,218); anti-mouse CCR2-PE (150609).

### In vitro CD8^+^ T cell cultures and cocultures with brain slice

Spleen was harvested from sham mice to prepare single cell suspensions as we described above. CD8^+^ T cells were isolated using mouse CD8a microbeads (Miltenyi Biotec, 130–117-044) according to the manufacturer’s instructions. Coculture System was established as described before [18], isolated CD8^+^ T cells in a transwell insert were incubated with brain slices in the lower chamber in culture media (RPMI 1640, 10% FBS, 1% penicillin/Strepromycin, 1 mM pyruvate sodium, 55 μm β-mercaptoethanol with the presence of soluble anti-CD3, anti-CD28 and IL-2) for 24 h. Then cells in the lower compartment were collected and stained with anti-mouse CD3e-FITC (1:200; BD Pharmingen, 553,062), anti-mouse CD8a-APC (1:200; BD Pharmingen, 553,035) on ice at 4 °C in the dark. CD3^+^ CD8^+^ T cells in the lower compartment were counted using Precision count beads (BioLegend, 424,902).

### Basic processing and clustering analysis of single-cell transcriptome data

Two single-cell RNA sequencing (scRNA-seq) datasets were downloaded from the Gene Expression Omnibus (GEO) database, including transcriptomic data of the SVZ neural stem cell niche in young and aged mice (PRJNA450425) and expression matrices of the spleen in young and aged mice (GSE132901). Basic processing and visualization of the scRNA-seq data were performed with the Seurat [19] package (v3.2.2) in R (v 3.6.3). Briefly, low-quality cells and doublets were filtered out based on the following criteria: (i) number of expressed genes was less than 200 or more than 2500 and (ii) the percentage of mitochondrial genes was more than 10%. The data were normalized to the total expression and log-transformed. The variable genes were detected using the FindVariableFeatures function with default parameters. Linear scaling was then applied and the mitochondrial contamination was removed using the ScaleData function. The batch effect was removed using the IntegrateData function. Principal component analysis was carried out on the scaled data, and the top 20 principal components were stored. Then, clusters were identified using the FindClusters function. Non-linear dimensional reduction methods including uniform manifold approximation and projection (UMAP) and t-distributed stochastic neighbor embedding (t-SNE) were used to visualize clustering results.

### Differentially expressed gene calculation

The Seurat FindAllMarkers and FindMarkers functions were employed to identify differentially expressed genes (DEGs) using the Wilcoxon rank sum test. Only genes with Bonferroni adjusted *P*-value < 0.05 and |log_2_(fold change) | > 0.1 were considered DEGs.

### Gene set enrichment analysis

Gene set enrichment analysis (GSEA) was carried out with the GSEA toolkit (v4.0.3, Broad Institute) following the published protocol [20]. Briefly, we generated a preranked gene list according to their log (fold change) and significance values. Five datasets (Hallmark pathways, Gene Ontology (GO) biological processes, KEGG, BioCarta, and Reactome) with in total 4495 gene sets were used as a reference. Then 1000 random permutations were performed to calculate the *P*-values. Significantly enriched gene sets were defined as gene sets with false discovery rate q-value < 0.05. Then, we used Cytoscape software (v3.7.2) and the AutoAnnotate app (v1.3.2) to cluster and annotate the gene sets.

### GO enrichment analysis

The online tool Metascape (http://metascape.org) was used for GO enrichment analysis [21]. All genes in the mouse genome were used as the enrichment background. Firstly, a set of DEGs was submitted. Metascape would return a set of statistically enriched terms. Then, the activation z-score of each term was calculated by the R package GOplot [22]. We defined significantly changed terms as those fulfilling *P*-value < 0.05 and an absolute z-score exceeding 2. Finally, the results were visualized with a chord chart or a dot plot using GOplot or prism software (v8.2.1, GraphPad Software Inc.).

### Pseudotime analysis

The Monocle R package (v2.14.0) was applied for pseudotime analysis [23–25]. Briefly, the top 250 highly variable genes were defined as the ordering genes. We reduced the dimensionality of data using the discriminative dimensionality reduction with trees algorithm with the reduceDimension function in Monocle. Then the cellular trajectory was constructed and the two-dimensional diffusion map was generated using plot_cell_trajectory and plot_genes_in_pseudotime functions.

### Cell–cell interaction analysis

We have developed the InterCellDB package for cell–cell interaction analysis of scRNA-seq data [26]. Briefly, the significant DEGs in each cluster were calculated as described before, and these genes were mapped to the mouse gene reference database, which was generated by collecting data from a variety of sources, including STRING, NCBI-gene, COMPARTMENTS, GO, and UniProt. Finally, matched gene pairs were visualized with the sankeyplot or dotplot function using networkD3 or InterCellDB. InterCellDB is publicly accessible as a R package in GitHub (https://github.com/ZJUDBlab/InterCellDB).

### Human tissue specimens

The radiologically healthy brain tissue sample was derived from patients undergoing brain surgery for epilepsy. Cerebrospinal fluid (CSF) was collected during preoperative lumbar puncture for patients older than 65 years with idiopathic normal intracranial pressure hydrocephalus (iNPH). After centrifugation (1500 g; 4 °C； 15 min), the supernatant was collected and stored at − 80 °C until further use. Sample collection and data analysis were approved by Institutional board of the Second Hospital affiliated to Zhejiang University (Protocol 2020–997).

### Primary human microglia cultures

The brain tissue was dissociated using the Adult Brain Dissociation Kit (Miltenyi Biotec) according to the manufacturer’s protocol. Microglia were isolated using the CD11b MicroBeads (Miltenyi Biotec) and then cultured (37 °C, 5% CO_2_) in Microglia Medium (ScienCell) with 5% fetal bovine serum,1% penicillin, 1% streptomycin and Microglia growth supplement for 7 days. At day 8, cultures were exposed to each individual CSF sample for 24 h. The exposure medium contained 25% CSF or PBS in microglia medium.

### RNA sequencing and data analysis

Total RNA from primary human microglia was isolated using TRIzol (Invitrogen, CA, United States) according to the manufacturer’s protocol. The RNA amount and purity of each sample was quantified using Nano Drop ND-1000 (NanoDrop, Wilmington, DE, USA) and the RNA integrity was assessed using the Agilent 2100 bioanalyzer. Sequence libraries were constructed according to the standard RNA-seq protocol, and 2 × 150 bp paired-end sequencing was performed with Illumina Novaseq 6000 (LC Bio) following the vendor’s recommended protocol. Cutadapt software was used to remove the reads that contained adaptor contamination. HISAT2 was used to align and map reads to the hg38 human reference genome. The mapped reads of each sample were assembled using StringTie. Then, all transcriptomes from the samples were merged to reconstruct a comprehensive transcriptome using perl scripts. After the final transcriptome was generated, StringTie and DEseq2 were used to estimate the expression levels of all transcripts. StringTie was used to perform expression level for mRNAs by calculating Fragment per Kilobase of transcript per Million mapped reads (FPKM). The differentially expressed genes (DEGs) were selected with fold change > 0.5 or fold change < − 0.5 and with statistical significance (Benjamini– Hochberg adjusted *p*-value < 0.05) by DESeq2 package [27].

### Statistical analysis

The scRNA-seq data were statistically analyzed using the Wilcoxon rank sum test. Statistical comparison of the means between the two groups was performed by using the student’s *t*-test or the Mann–Whitney U test. Multiple comparisons were analyzed by one-way analysis of variance (ANOVA) followed by the Bonferroni multiple comparison test. All statistical analyses were performed with R (v.3.6.3) or GraphPad Prism (v.8.2.1). Statistical significance was defined as *P* ≤ 0.05.

## Results

### Peripheral T cells infiltrate the aged SVZ and remodel the brain microenvironment

**Fig. 1:**
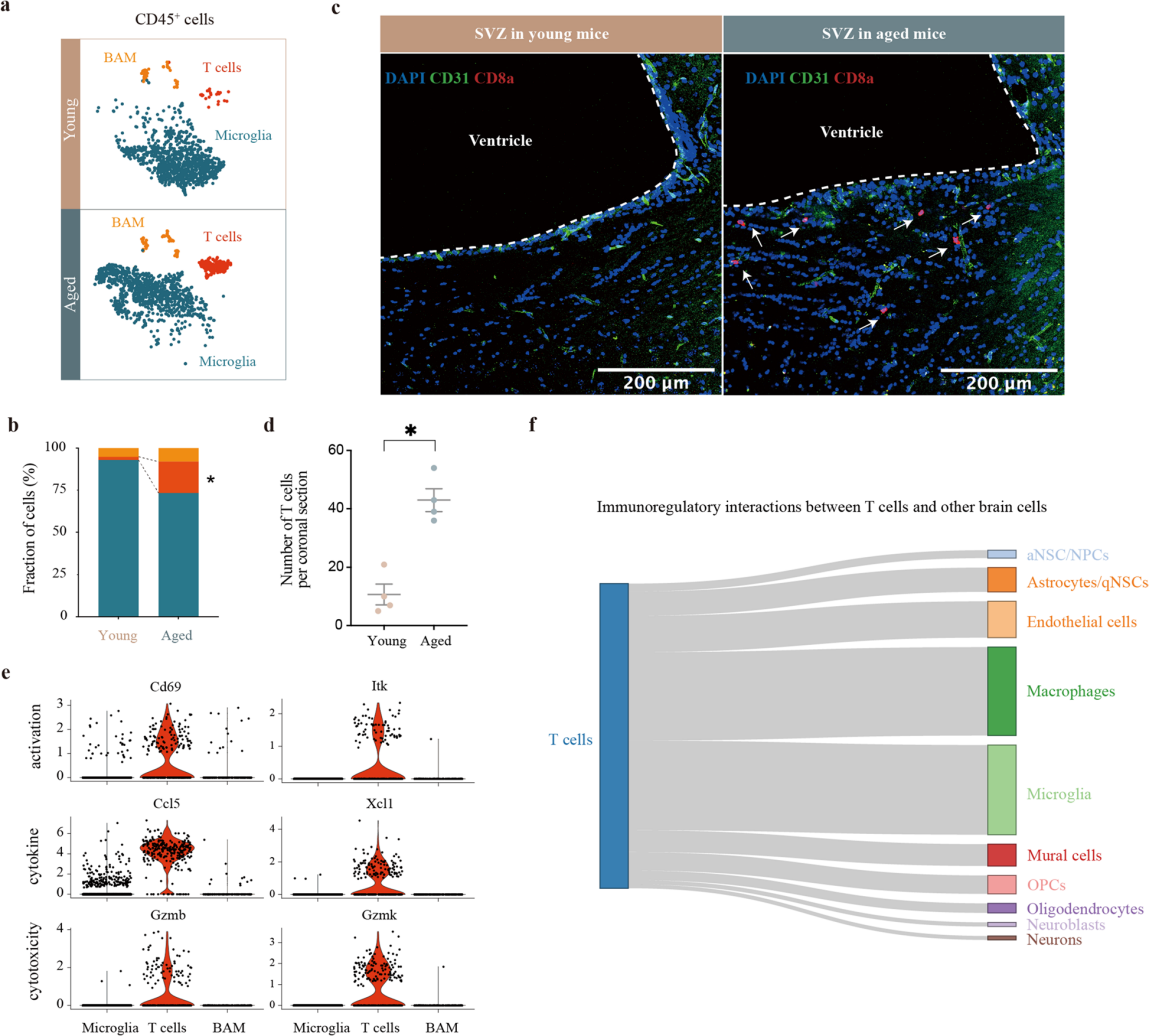
T cells infiltrate the old neurogenic niches and affect the brain microenvironment. **a** t-SNE projection of the CD45^+^ cells in the SVZ from three young (3 months old) and three old (28–29 months old) mice. Cell types are color-coded and annotated based on the transcriptomic profiles. **b** Bar plot showing the fraction of cells associated with each cell type in the young and aged SVZ. *P ≤ 0.05 by one-tailed Wilcoxon rank sum test. **c** Representative confocal microscopic images of young and old SVZs stained for CD31 and CD8a. Nuclei are labeled with DAPI. Scale bar: 200 μm. **d** Number of CD8^+^ T cells per coronal section in four young (6–8 weeks old) and four aged (18 months old) male mice. *P = 0.0286, Mann–Whitney test, two sided. Data are shown as mean ± s.e.m. **e** Violin plots showing expression of various T cell activation- (*Cd69*, *Itk*), cytokine release- (*Ccl5*, *Xcl1*), and cytotoxicity-related (*Gzmb*, *Gzmk*) genes in each of three distinct clusters. **f** Sankey diagram depicting the interaction between T cells and resident cells in the SVZ based on the Reactome term *immunoregulatory interactions between a lymphoid and a non-lymphoid cell* (R-HSA-198933). The proportional flow represents the number of gene pairs. Gene pairs are listed in Supplementary Table 2

Brain aging has been reported to be accompanied by chronic inflammation. To investigate the molecular and cellular alterations of the brain immune microenvironment during normal aging, we explored the large scale transcriptomic dataset (PRJNA450425) of single cells derived from three young (3 months old) and three aged (28–29 months old) mouse SVZs (Supplementary Fig. 1a). On the basis of quality control, 13,760 cells were used for unsupervised clustering analysis (Supplementary Fig. 1b), and 2800 CD45^+^ cells were extracted for downstream analysis (Supplementary Fig. 1c). Sub-clustering analysis revealed microglia, T cells, and CNS border-associated macrophages were the major immune cells in the SVZ **(**Fig. 1a**,** Supplementary Fig. 1d). Notably, the T cells were almost exclusively derived from aged mice, and the proportion of T cells was significantly larger in aged SVZ **(**Fig. 1b). We further identified the T cells as CD3^+^CD8^+^CD4^−^ by exploring the expression of marker genes, including *Cd3e*, *Runx*, *Cd4*, and *Cd8a* (Supplementary Fig. 1e). Immunofluorescence staining of CD31 and CD8a confirmed the obvious age-related T cells infiltration in the SVZ in both male and female mice **(**Fig. 1c-d, Supplementary Fig. 1f).

The infiltrated T cells highly expressed genes related to T cell activation (*Cd69*, *Itk*), cytokine release (*Ccl5*, *Xcl1*), and cytotoxicity (*Gzmb*, *Gzmk*) **(**Fig. 1e). We further performed functional enrichment analysis to clarify the role of the infiltrated T cells. A total of 335 GO terms were enriched (z-score ≥ 2, adjusted *P*-value < 0.05) (Supplementary Table 1). Top functional terms were T cell receptor (TCR) signaling, T cell activation, and immunoregulatory interactions between a lymphoid and a non-lymphoid cell (Supplementary Fig. 1 g). These results suggested an activated phenotype of the infiltrated T cells, which might have impact on the brain microenvironment. We further applied the InterCellDB toolkit to determine the effect of T cells on resident cells in the brain. First, we focused on the genes related to the Reactome term *immunoregulatory interactions between a lymphoid and a non-lymphoid cell* (R-HSA-198933). A widespread effect of the infiltrated T cells was identified. Microglia (432gene pairs), macrophages (426 gene pairs) and endothelial cells (175 gene pairs) were the main resident cells in the SVZ in communications with the infiltrated T cells (Fig. 1f, Supplementary Table 2). Second, we performed intercellular network analysis based on the cytokines released by T cells. This analysis identified C*cl4*, C*cl5*, I*fng*, Xcl1, and Fasl as key cytokine-coding genes affecting the brain microenvironment since they showed effects on almost all resident cells in the SVZ (Supplementary Fig. 1 h, Supplementary Table 3).

### Aged microglia release chemokines to recruit circulating CD8^+^ memory T cells

**Fig. 2:**
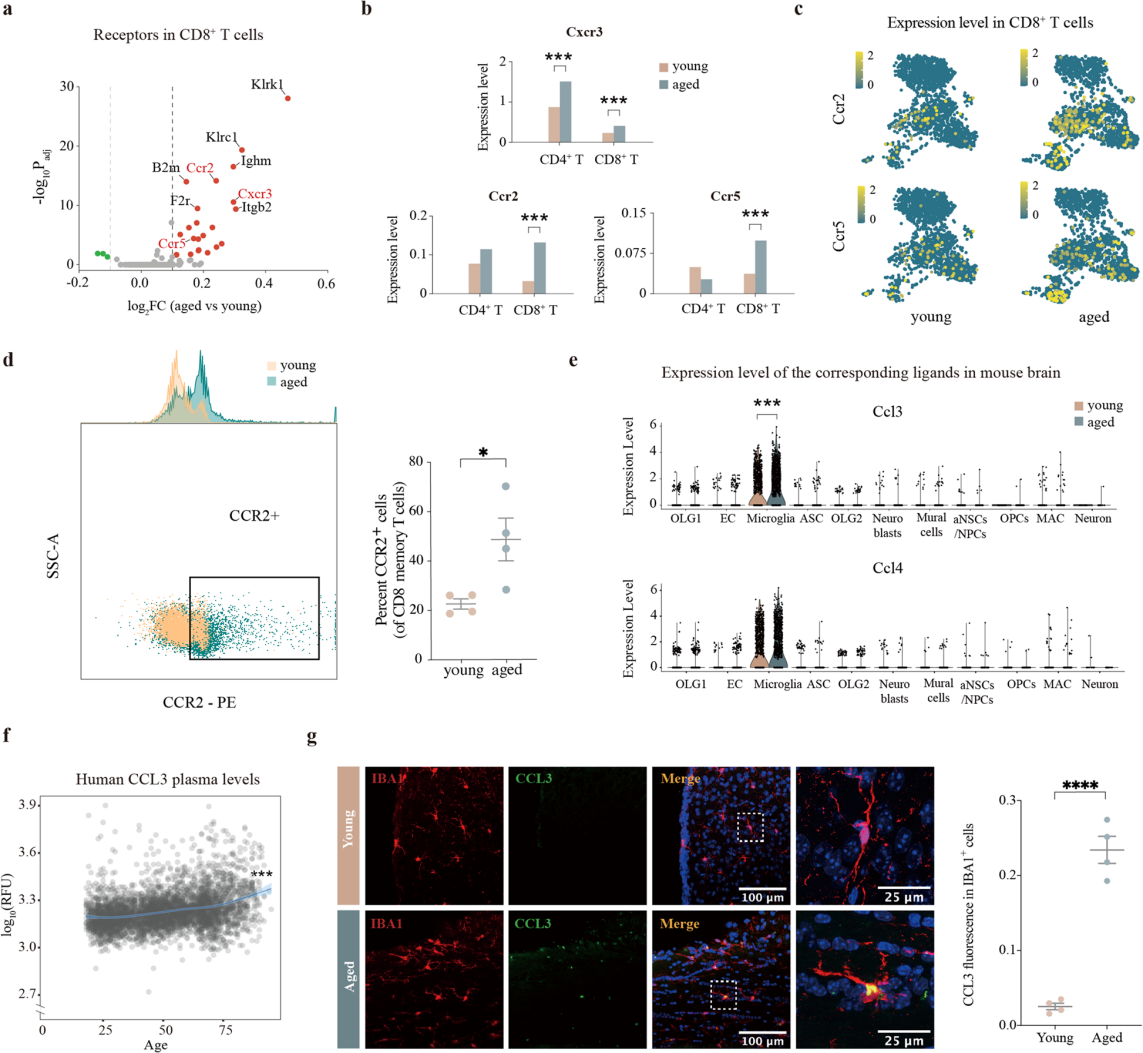
Transcriptomic alteration of chemokines and receptors in aged microglia and peripheral T cells. **a** Volcano plot depicting the differentially expressed receptor genes in aged CD8^+^ T cells compared to young CD8^+^ T cells. Differentially expressed genes (DEGs; |log_2_(fold change)| > 0.1, Bonferroni adjusted *P*-value < 0.05) were colored (red for upregulated DEGs and green for downregulated DEGs). **b** Bar plot showing the expression levels of *Cxcr3*, *Ccr2*, and *Ccr5* in CD4^+^ T cells and CD8^+^ T cells from young and old mice. ***Bonferroni adjusted *P*-value ≤0.001 by Wilcoxon rank sum test. **c** Expression profiles of *Ccr2* and *Ccr5* in both young and aged CD8^+^ T cells are shown using the UMAP visualization approach. **d** Protein expression of CCR2 by splenic CD8^+^ memory T cells of four young (6–8 weeks old) and four aged (18 months old) male mice. *P = 0.0264, two-tailed Student’s t test. Data are shown as mean ± s.e.m. **e** Violin plots showing expression of *Ccl3* and *Ccl4* in young and old mouse brains. *** Bonferroni adjusted *P*-value ≤0.001 by Wilcoxon rank sum test. **f** Dot plot showing human CCL3 plasma levels. Linear regression is depicted with the colored line. Type II sums of squares were calculated and tested using the F-test. Q-values were estimated using the Benjamini–Hochberg approach. ***P = 4.36 × 10^− 33^; q = 1.14 × 10^− 31^). **g** Left: Representative confocal microscopic images of young and old SVZs stained for CCL3 and IBA-1. Nuclei are labeled with DAPI. Scale bars: 100 μm or 25 μm. Right: Quantification of CCL3 fluorescence in IBA1^+^ cells in four young (6–8 weeks old) and four aged (18 months old) male mice. ****P < 0.0001, two-tailed Student’s t test. Data are shown as mean ± s.e.m.

Peripheral T cells undergo tremendous changes with age [28]. In order to determine whether these changes contribute to the T cell infiltration in the SVZ, we analyzed another published single-cell transcriptomic dataset (GSE132901) of splenic T cells [29] (Supplementary Fig. 2a). A total of 30,168 cells from four young (29–34-wk-old) and three aged (88–93-wk-old) mice were included in unsupervised clustering analysis, and a CD8^+^ T cell cluster containing 3318 cells was extracted for sub-clustering and further downstream analysis (Supplementary Fig. 2b). A volcano plot displayed all receptor genes whose expression changed significantly with age in CD8^+^ T cells. Three chemokine receptor genes, *Cxcr3*, *Ccr2*, and *Ccr5*, were significantly upregulated in aged CD8^+^ T cells, which were possibly associated with the recruitment of CD8^+^ T cells (Fig. 2a). We noted that *Cxcr3* was robustly upregulated in both CD4^+^ and CD8^+^ T cells, while *Ccr2* and *Ccr5* were upregulated specifically in CD8^+^ T cells (Fig. 2b), which indicated that *Ccr2* and *Ccr5* may account for the selective CD8^+^ T cell infiltration in the aged SVZ. Interestingly, *Ccr2* and *Ccr5* showed a remarkable heterogeneity in gene expression in CD8^+^ T cells in both young and aged mice (Fig. 2c).

Unsupervised sub-clustering analysis was applied to explore the heterogeneity of CD8^+^ T cells. The analysis of 3318 CD8^+^ T cells with UMAP revealed four distinct cell types (Supplementary Fig. 2b). Characterization of markers in these clusters (Supplementary Fig. 2c) identified them as naive T cells, central memory T cells (Tcm), effector memory T cells (Tem), and interferon response T cells (Supplementary Fig. 2b). The *Ccr2* and *Ccr5* genes were exclusively expressed in memory T cells (Fig. 2c), indicating their propensity to be attracted to the brain. Flow cytometry experiments confirmed the remarkable increase of CCR2^+^ CD8 memory T cells in both male and female mice (Fig. 2d, Supplementary Fig. 2d-e).

We then screened the expression levels of the genes encoding their corresponding ligands in the mouse brain. *Ccl3* and *Ccl4* were expressed specifically in microglia, and *Ccl3* was significantly upregulated in aged microglia (Fig. 2e). In order to clarify whether CCL3 plays a role in trafficking of CD8^+^ T cells to the aged brain, we downloaded the Aging Plasma Proteome data from a previous study [30]. This dataset contains data on 2925 plasma proteins from 4263 subjects ranging from young adults to nonagenarians. As expected, the plasma CCL3 level increased significantly during normal aging (Fig. 2f). Immunofluorescence staining was performed to verify the expression of CCL3 and CCL4 in microglia. We observed strongly increased staining of CCL3 and CCL4 in old microglia compared with their younger counterparts (Fig. 2g, Supplementary Fig. 2f-g).

### Various age-related transcriptomic changes of brain endothelial cells from different arteriovenous zones

**Fig. 3:**
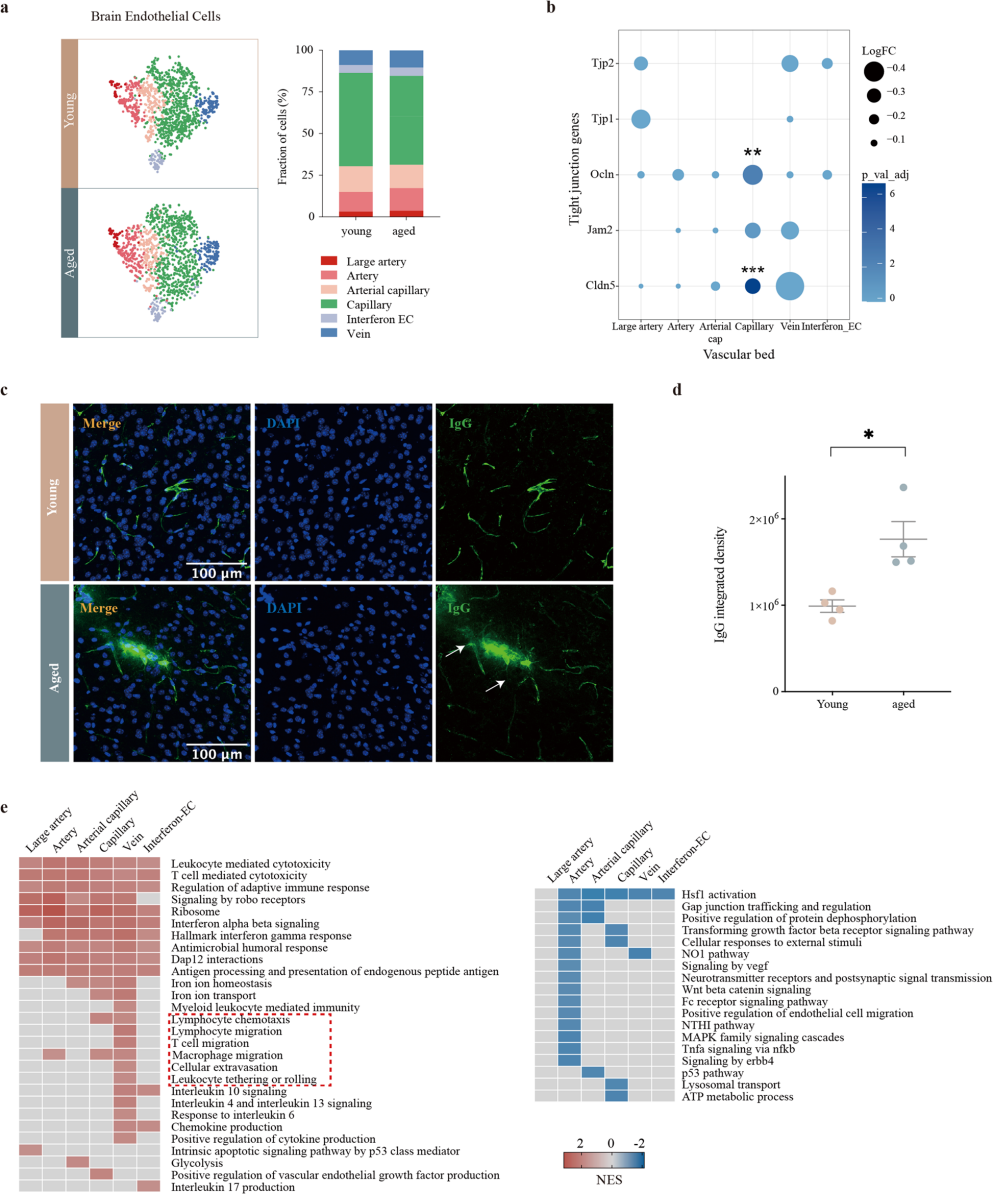
Age-related changes of BECs from different arteriovenous zone. **a** t-SNE projection of brain endothelial cells in the SVZ from three young (3 months old) and three old (28–29 months old) (left). Cell types are color-coded and annotated based on the transcriptomic profiles. Bar plot showing the fraction of cells associated with each cell type from both young and aged mice (right). **b** Dot plot showing the expression of tight junction genes across different vascular segments. Node size represents the magnitude of DEGs (log_2_(fold change)) and node color represents adjusted *P*-values. **Bonferroni adjusted *P*-value ≤0.01, ***Bonferroni adjusted *P*-value ≤0.001 by Wilcoxon rank sum test. **c** Representative confocal microscopic images of young and old mouse brains stained for IgG. Nuclei are labeled with DAPI. Scale bars: 100 μm. **d** Quantitative analysis of IgG extravascular deposits in four young (6–8 weeks old) and four aged (18 months old) male mice. *P = 0.0117, two-tailed Student’s t test. Data are shown as mean ± s.e.m. **e** Heatmap of GSEA showing part of the significant (FDR q-value < 0.05) aging-related pathways across vessel segments. Numbers in the legend represent normalized enrichment score (NES); positive values indicate upregulation and negative values indicate downregulation

BECs serve as relay stations between the circulation and the brain. Immunofluorescence staining showed CD31 and CCL3 were partially co-localized in the aged SVZ (Supplementary Fig. 3a), indicating that aged BECs may also contribute to the T cell recruitment. Then, we extracted and re-clustered the BECs of the SVZ for further downstream analysis (Supplementary Fig. 3b–c). Unsupervised sub-clustering analysis revealed seven distinct clusters (clusters E0–E6, Supplementary Fig. 3c). Notably, the trajectory constructed by Monocle pseudotime analysis was strongly associated with the arteriovenous axis. Known arterial (*Fbln5*) and venous (*Nr2f2*) markers peaked at opposite ends of the trajectory and the capillary (*Rgcc*) markers summited in the middle of the trajectory (Supplementary Fig. 3d). Then, we visualized each cluster of BECs on the trajectory (Supplementary Fig. 3e), and redefined them as large artery (E6), artery (E3), arterial-capillary (E2), capillary (E0, E1), vein (E4), and interferon-EC (E5) based on their transcriptomic profiles (Supplementary Fig. 3e, Fig. 3a). In addition, we identified 154 artery-specific markers, 22 capillary-specific markers, and 57 vein-specific markers (Supplementary Fig. 3f; Supplementary Table 4).

The cellular composition was similar across young and aged BECs (Fig. 3a). Nonetheless, we observed generally downregulation of tight junction component-encoding genes across the vascular network (Fig. 3b). Previous studies have reported that aged BECs downregulate the expression of *Cldn5* (encoding claudin 5) and *Ocln* (encoding Occludin) [31, 32]. We observed a significant downregulation of *Cldn5* and *Ocln* in capillary BECs, but not in arterial or venous BECs (Fig. 3b, Supplementary Fig. 3 g). In addition, we found obvious extravasation of IgG in the aged mouse brain (Fig. 3c-d). Together, these data indicated that tight junctions were dysfunctional in brain microvascular endothelial cells (BMECs).

To investigate how aging may commonly and differentially affect gene expression of BECs from different vascular beds, GSEA analysis was performed to assay aging-related pathways in each BEC subtype (Fig. 3e). Consistent with recent reports [14, 31, 33], we found an upregulation of immune/cytokine signaling (adaptive immune response, interferon signaling, and antigen presentation) and ribosome biogenesis for all vascular segments (Fig. 3e). In addition, a significant downregulation of Wnt–β-catenin signaling was observed in the arterial segment, which had been identified as a key regulator of BBB maintenance [34]. Aged capillaries downregulated “ATP metabolic process”, implicating dysfunctional energy metabolism in aged BMECs. Notably, upregulation of “T cell migration-related process” was specifically observed in aged venous BECs (Fig. 3e), which implicated the vascular bed dependence of T cell infiltration in the aged SVZ.

### Venous BECs upregulate adhesion molecules and promote T cell infiltration in the aged SVZ

**Fig. 4:**
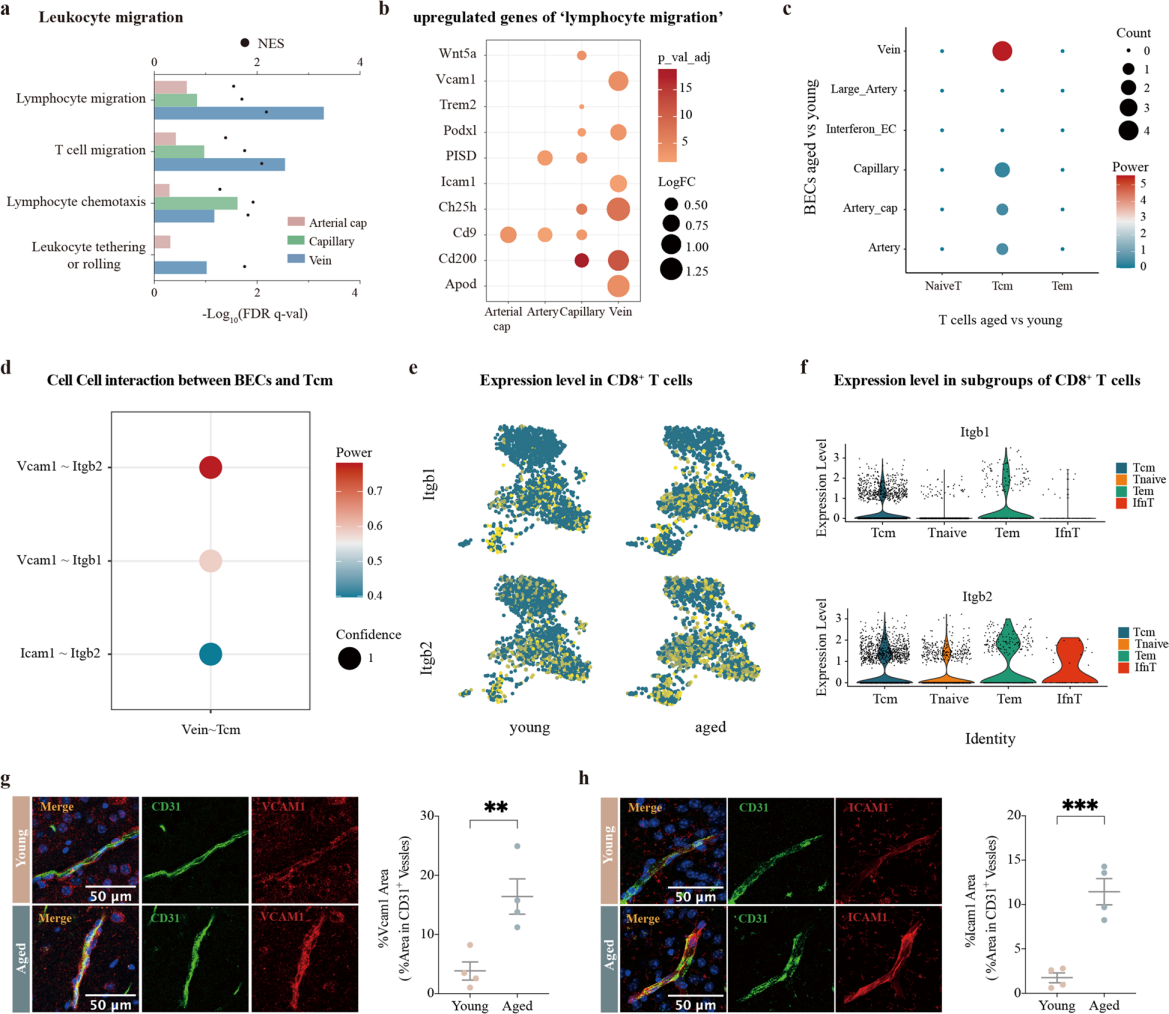
Cell–cell interaction between venous BECs and Tcm. **a** The activation status of biological processes related to leukocyte migration was assessed by calculating their NES using GSEA. Shown are the biological processes associated with leukocyte migration that were predicted to be activated in arterial capillary, capillary, and vein clusters. **b** Dot plot showing the upregulated genes in the term *lymphocyte migration* across different vascular segments. Dot size means the power of gene pairs by summing up the log_2_(fold change) values of participating genes. Dot color means the aggerated confidence for gene pairs. **c** Dot plot depicting the interaction between T cells and BECs from different vascular segments. Node size represents the count of interaction pairs and node color represents the power of interaction pairs. **d** Dot plot showing the gene pairs between venous BECs and central memory T cells. Gene pairs with *p*-value < 0.05 in permutation test are fetched. Dot color represents the power of gene pairs by multiplying the expression levels of participating genes. **e** Expression profiles of *Itgb1* and *Itgb2* in both young and aged CD8^+^ T cells are shown using the UMAP visualization approach. **f** Violin plots showing expression of *Itgb1* and *Itgb2* in young and aged CD8^+^ T cells. **g**–**h** Representative confocal microscopic images and quantification of VCAM1 **(g)**, ICAM1 **h** colocalization with CD31 in four young (6–8 weeks old) and four aged (18 months old) male mice. Nuclei are labeled with DAPI. Scale bars: 50 μm. **P = 0.0096 (g), ***P = 0.0008(h), two-tailed Student’s t test. Data are shown as mean ± s.e.m.

To further clarify the mechanism by which T cells infiltrate the aged SVZ and the exact location, we deeply examined all biological processes associated with leukocyte migration among different vascular segments (Fig. 4a). This functional cluster was predicted to be strongly activated in venous BECs in the aged SVZ. Subsequently, we extracted all upregulated DEGs involved in *lymphocyte migration* and depicted a dot plot to display the relationship between these genes and the vascular bed (Fig. 4b). Numerous key genes were significantly upregulated in aged venous BECs, including *Vcam1*, *Podxl*, *Icam1*, *Ch25h*, *Cd200*, and *Apod* (Fig. 4b). Notably, two genes encoding critical adhesion molecules of endothelial cells, *Vcam1* and *Icam1*, were specifically upregulated in venous BECs (Fig. 4b). These two molecules play an important role in mediating the firm adhesion of leukocytes to endothelial cells. Then, we applied the InterCellDB toolkit to explore cell–cell interactions between T cells and BECs (Fig. 4c). Strong interactions between venous BECs and Tcm were observed (Fig. 4c). We further explored gene pairs that mediate cell migration between BECs and Tcm. The results showed that the *Vcam1*–*Itgb2*, *Vcam1*–*Itgb1* and *Icam1*–*Itgb2* pairs were relatively specific for Vein–Tcm communication (Fig. 4d). Notably, *Itgb1*, *Itgb2*, and *Ccr2* were selectively expressed in aged memory T cells (Fig. 4e–f**,** Fig. 2c). Finally, expression of VCAM1 and ICAM1 in cells immunoreactive for CD31, a marker of brain endothelial cells, was significantly upregulated in the aged SVZ (Fig. 4g–h).

### Aged microglia promote expression of adhesion molecules on venous BECs

**Fig. 5:**
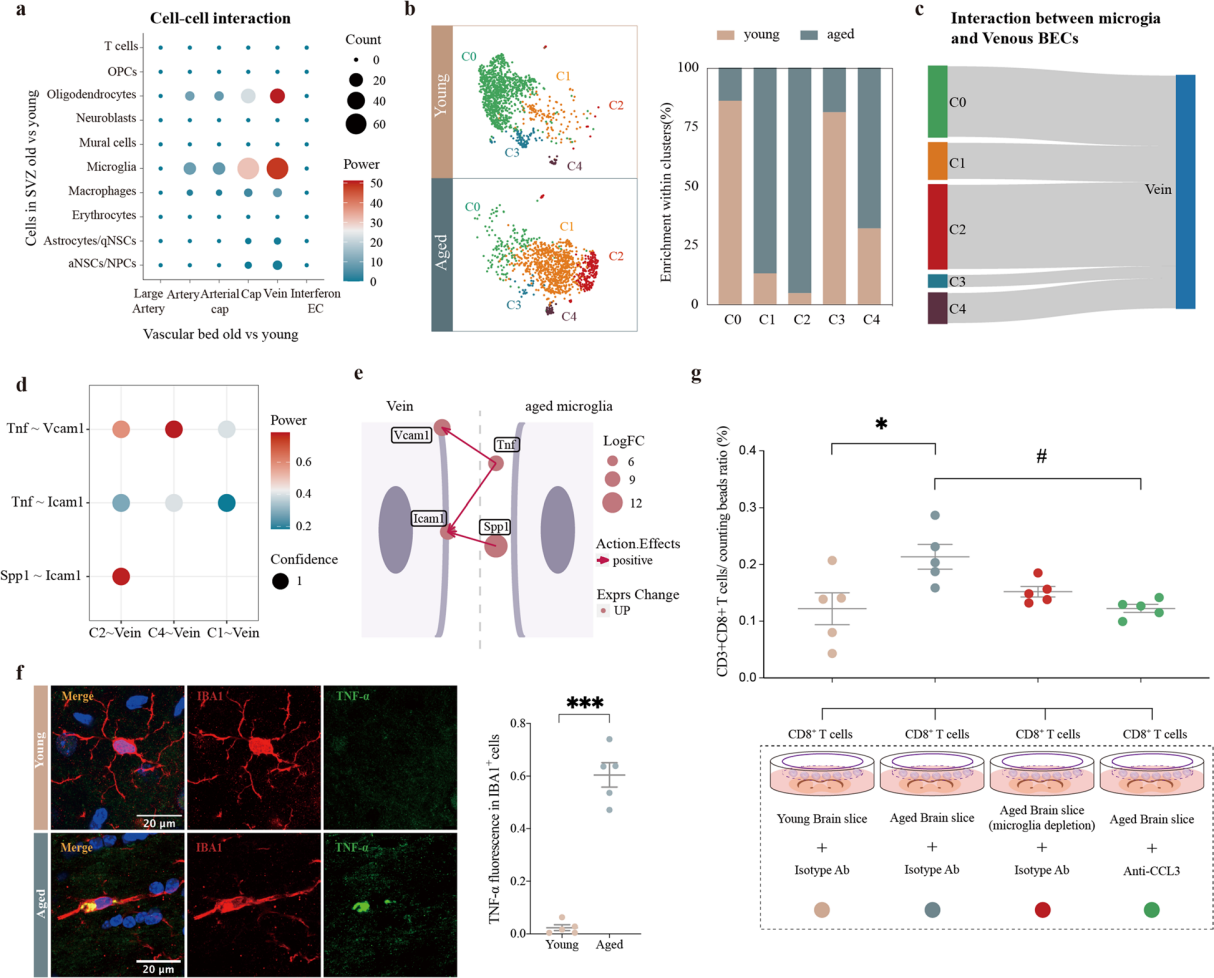
Cell–cell interactions between aged microglia and venous BECs. **a** Dot plot depicting the interaction of “leukocyte migration” between cells in the SVZ and BECs from different vascular segments. Node size represents count of interaction pairs and node color represents power of interaction pairs. **b** t-SNE projection of microglia in the SVZ from young and aged mice (left). Cell types were color-coded and annotated based on the transcriptomic profiles. Bar plot showing the fraction of cells associated with each cell type from both young and aged mice (right). **c** Sankey diagram depicting the interactions between microglia and venous BECs. The proportional flow represents the number of gene pairs. **d** Dot plot showing the gene pairs between venous BECs (old vs. young) and microglia. Gene pairs with p-value < 0.05 in permutation test are fetched. Dot color represents the power of gene pairs by multiplying the expression levels of participating genes. **e** Cell plot showing interaction pairs between microglia and vein. The vivid two-cell graph was generated by the InterCellDB package using default settings. **f** Left: Representative confocal microscopic images of young and old mice brains stained for TNF-α and IBA-1. Nuclei are labeled with DAPI. Scale bars: 20 μm. Right: Quantification of TNF-α fluorescence in IBA1^+^ cells in four young (6–8 weeks old) and four aged (18 months old) male mice. ***P = 0.0008, two-tailed Student’s t test. Data are shown as mean ± s.e.m. **g** CD8^+^ T cells in inserts were cocultured with young brain slice, aged brain slice, microglia-depleted aged brain slice and aged brain slice with anti-CCL3 for 24 h. Cells in the lower compartment were collected for flow cytometry analysis. Each dot represents one mice. *Adjusted-*P* = 0.0155(young brain slie vs aged brain slice), # Adjusted-*P* = 0.0161(aged brain slice vs aged brain slice + anti-CCL3), One-way ANOVA. Data are shown as mean ± s.e.m.

To further investigate the cause of transcriptome changes in venous BECs, we analyzed cell–cell interactions between brain resident cells and venous BECs (Fig. 5a). There is a strong impact of aged microglia on the function of “leukocyte migration” in venous BECs (Fig. 5a). Thus, we compared the transcriptomic landscape of microglia between young and aged mice (Supplementary Fig. 4a–b). Five distinct clusters of microglia were identified in both young and aged mice (C0–C5, Fig. 5b). Interestingly, microglia from aged mice were most enriched in clusters 1, 2 and 4, whereas those from young mice were more frequently found in clusters 0 and 3 (Fig. 5b). There were strong communications between aged microglia (C1 and C2) and venous BECs (Fig. 5c). Thus, we performed differential expression analysis between cluster 2 and cluster 0 to determine the transcriptomic changes induced by aging in homeostatic microglia (Supplementary Fig. 4c). We identified 405 upregulated DEGs, the functional implications of which were further explored by GO enrichment analysis. Five functional clusters were significantly overrepresented in aged microglia, among which the largest was associated with the inflammatory response and cytokine production (Supplementary Fig. 4d). We further annotated the relationship between specific genes and biological processes in the *inflammatory response and cytokine production* functional cluster (Supplementary Fig. 4e). Numerous key genes upregulated in aged microglia, including *Apoe*, *Spp1*, *Cd74*, *Ccl3*, *Mif*, *Ccl4*, and *Tnf*, were involved in at least two subcategories of the functional cluster (Supplementary Fig. 4e).

We further explored whether cytokines secreted by microglia could activate venous BECs in the aged SVZ. We applied the InterCellDB toolkit and found several gene pairs between aged microglia and venous BECs (Fig. 5d). Gene pairs including *Tnf*–*Vcam1*, *Tnf*–*Icam1*, and *Spp1*–*Icam1* were specific with positive effects (Fig. 5e). TNF-α is a crucial pro-inflammatory cytokine with elevated expression during normal aging [35]. Then We performed double-label immunofluorescence staining and observed a significantly higher number of TNF-α^+^ microglia in both male and female aged mice (Fig. 5f**,** Supplementary Fig. 4f).

Together, these results indicated that aged microglia might shift towards a pro-inflammation and chemotactic state and recruit peripheral T cells into aged brain. To confirm these observations obtained by transcriptomic analysis, we established a coculture system to further dissect the impact of aged microglia on CD8^+^ T cells. CD8^+^ T cells were isolated from mouse spleens using magnetic-activated cell sorting and then cocultured with brain slices in the lower compartment for 24 h. Cells in the lower compartment were then collected for flow cytometry analysis (Supplementary Fig. 4 g). The results revealed a significantly higher number of CD8^+^ T cells entering the lower compartment when co-cultured with aged brain slices compared to co-cultured with young brain slices **(**Fig. 5g**)**. However, depleting microglia with PLX5622 or blocking CCL3 could partially reduce the number of CD8^+^ T cells recruited by aged brain slice **(**Fig. 5g**)**. These results support a critical contribution of aged microglia to CD8^+^ T cells recruitment in aged mouse brain.

### CSF from the elderly promotes the transition of human microglia to a chemotactic phenotype in vitro

**Fig. 6:**
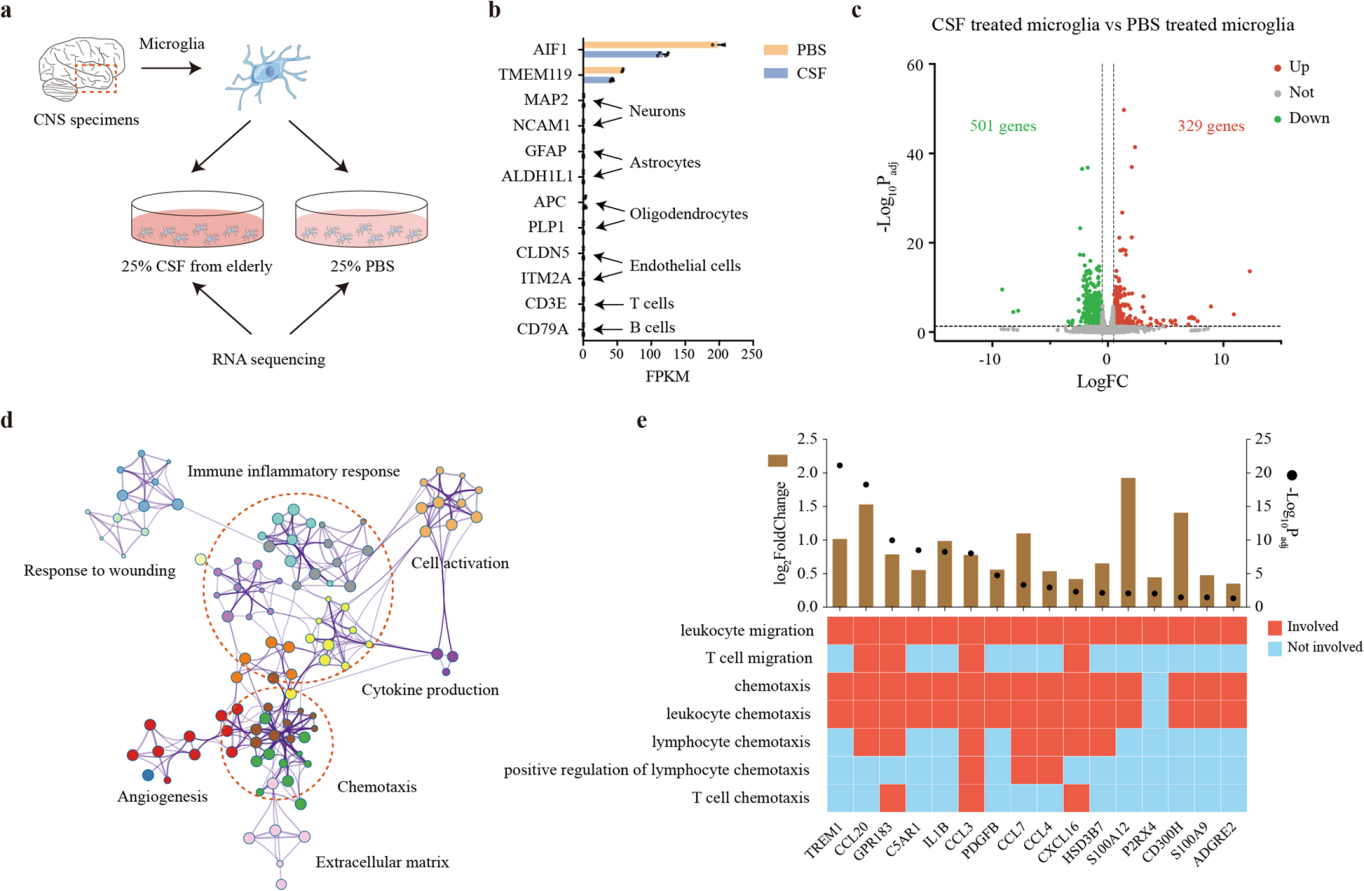
Transcriptional regulation of chemotaxis-related genes in human microglia after treating with CSF from the elderly. **a** Sorted primary human microglia were cultured with CSF from the elderly (*n* = 4) or PBS (*n* = 2) for 24 h and then collected for RNA sequencing. **b** mRNA expression levels of microglia (AIF1,TMEM119), neurons (MAP 2, NCAM1), Astrocytes (GFAP, ALDH1L1), Oligodendrocytes (APC, PLP1), Endothelial cells (CLDN5, ITM2A), T cells (CD3E) and B cells (CD79A) marker genes. **c** Volcano plot depicting the differentially expressed genes in CSF treated microglia compared to PBS treated microglia. Differentially expressed genes were colored (red for upregulated DEGs and green for downregulated DEGs). **d** Functional enrichment analysis was performed using Metascape on the upregulated DEGs in (c). The significantly overrepresented (*P* < 0.01) terms were grouped into color-coded clusters based on their membership similarities and rendered as a network plot. Each node represents an enriched term, and one representative term is shown for each cluster. Terms with a similarity > 0.3 are connected by edges. **e** Expression profiles of DEGs in CSF treated microglia related to leukocyte recruitment pathways

Next, we investigated the similarity in expression profiles and associated biological properties between human and mouse primary microglia during normal aging. To obtain primary human microglia, we collected radiologically healthy CNS specimens from patients undergoing brain surgery for removal of epileptic foci. Then the tissues were dissociated and sorted using magnetic-activated cell sorting for cells positive for CD11b, which were then transferred into 6-well plates. After exposed to each individual CSF sample or PBS for 24 h, the cells were collected for RNA sequencing (Fig. 6a). The sorted human microglia expressed known microglial genes such as AIF1, TMEM119 (Fig. 6b). In contrast, the well-established markers of neurons (MAP 2, NCAM1), astrocytes (GFAP, ALDH1L1), oligodendrocytes (APC, PLP1), endothelial cells (CLDN5, ITM2A), T cells (CD3E), and B cells (CD79A) were all expressed at low levels (Fig. 6b). Next, to determine the transcriptomic changes of microglia induced by the CSF from the elderly, we applied the R package DEseq2 to calculate the DEGs, which contains 329 upregulated genes and 501 downregulated genes (Fig. 6c, Supplementary Table 5). To elucidate the functional alterations in the microglia treated with CSF from the elderly, GO enrichment analysis was performed on the upregulated DEGs by an online tool Metascape. Immune inflammatory response, cytokine production, cell activation and chemotaxis accounted for the majority of enriched GO terms (Fig. 6d), which showed remarkable similarity with the results of aging mouse microglia (Supplementary Fig. 4d). Since we identified chemotaxis as an important functional alternation of aging mouse microglia, we further explored the pathways related to leukocyte migration and chemotaxis. There were many significantly upregulated chemokines including CCL3 and CCL4 which were involved in three or more GO functional terms (Fig. 6e), indicating their critical roles in recruiting peripheral immune cells.

## Discussion

In this study, we applied intercellular network analysis of single-cell transcriptomic data from young and aged mice. Major findings of our study include that (i) peripheral T cells infiltrate the neurogenic niche during normal aging and extensively affect the brain microenvironment; (ii) aged microglia release CCL3 to recruit CD8^+^ memory T cells; and (iii) aged microglia shift towards a pro-inflammatory state and release TNF-α to activate venous BECs, which specifically upregulate VCAM1 and ICAM1 and promote the transendothelial migration of T cells.

Emerging evidence indicates that the immune privilege in the aging CNS is compromised [2, 36]. CD8^+^ T cells and natural killer cells were reported to accumulate in the aging brain [1, 5, 37]. In our study, we identified a considerable increase of CD8^+^ T cells in the aged SVZ using single-cell transcriptomic analysis and immunofluorescence. Several studies have emphasized the harmful role of the infiltrated T cells. T cells in the aged brain were reported to be detrimental for neural stem cells function by inducing interferon-γresponse [5]. Accumulation of CD8^+^ T cells drives axon degeneration in the normal aging mouse CNS and contributes to age-related cognitive and motor decline [1]. Similarly, we found that the infiltrated T cells express markers associated with TCR activation and interactions with non-lymphoid cells. They were involved in a complex intercellular network and showed significant interactions with microglia, macrophages, endothelial cells, and oligodendrocytes. Furthermore, our data showed that several cytokines they release, such as C*cl4*, C*cl5*, X*cl1*, I*fng*, F*lt3l,* and L*tb*, widely affect various resident cells in the aged brain.

The major objective of this study was to explore transcriptomic alterations in the brain microenvironment when hematogenous T cells enter the brain through intercellular analysis, as there are only few leukocytes in the brain participating in immune surveillance in a healthy state. A critical issue that needs to be addressed is whether T cells enter the aged brain passively or actively. Our results suggest that the age-related T cell infiltration is primed by microglia. We found that hematogenous CD8^+^ T cells undergo tremendous changes during normal aging, which corroborated evidence from previous studies [28, 38]. While the upregulation of C*cr2* and C*cr5* was specifically found in CD8^+^ memory T cells, their corresponding ligand CCL3, an important chemokine in the migration of effector T cells during CNS infection [39], showed an age-related upregulation in human plasma and in microglia. These results implicated that microglia actively recruit CD8^+^ memory T cells during normal aging. These findings are in line with previous reports, in which the TCR repertoire of aged brain T cells were identified to be clonally expanded and different from that of aged blood, supporting the antigen-driven infiltration hypothesis [5].

Endothelial cells are major participants in and regulators of the inflammatory response [40]. Their interaction with circulating leukocytes is a critical step in pathogenesis of inflammation reactions. A plethora of studies showed that adhesion molecules and chemokines play critical roles in T cell migration across the BBB [41, 42]. However, no studies have thoroughly investigated the mechanisms underlying age-related T cell infiltration across the BBB. Single-cell transcriptomic analysis allows us to identify endothelial cell subtypes along the arteriovenous axis [43, 44]. According to the reported transcriptomic profiles of different vascular zones, we classified the BEC transcriptome into six subclusters. Consistent with a previous study [14], we found that aging induced distinct transcriptomic alterations across the vascular bed. T cell immune surveillance mainly occurs in the perivascular space of postcapillary venules [8]; correspondingly, we found that biological processes related to T cell migration were specifically upregulated in venous BECs with age. Using cell–cell interaction analysis, we identified *Vcam1* and *Icam1* as key genes mediating age-related T cells transendothelial migration by firm adhesion and spreading of leukocytes. Although studies focused on the normal aging brain remain limited, Yousef et al. have shown that blocking VCAM1 could reverse microglial reactivity and cognitive deficits in the brain of aged mice [45]. Lastly, our data revealed that aged microglia shift towards a pro-inflammatory state and significantly upregulate TNF-α, which could evoke an inflammatory response of BECs and upregulate the expression of VCAM1 and ICAM1 [46]. All of these findings from our present study indicate that the pro-inflammatory cytokines released by aged microglia are major contributing factors affecting the upregulation of adhesion molecules on venous BECs.

Several limitations of the present study should be noted. First, our findings are mainly based on the analysis of single-cell transcriptomic data from small number of mice, while changes in the transcriptome do not always dictate molecular alterations at the protein or functional level. In this study, we have verified several changes at the protein and functional levels including the infiltration of CD8^+^ T cells, IgG extravasation, and CCL3, CCL4, VCAM1, ICAM1, and TNF-α protein expression. Nevertheless, further studies are needed to verify the functional implication of transcriptional alterations derived from this report. Second, the present research only focused on the establishment of firm adhesion between T cells and BECs. The mechanisms by which T cells cross the endothelium and the glia limitans during normal aging still need to be further studied.

The absence of transcriptomic analysis in aged female mice is an important flaw in this paper, given the salient sex differences in the aging process [47]. To reduce the gender bias on our conclusions, we validated our main results in female mice. First, we found an obvious age-related T cells infiltration in the SVZ in female mice. And a remarkable increase of CCR2^+^ CD8 memory T cells was also identified in female mice. Besides, we found aged microglia in female mice also transit to a chemotactic and pro-inflammation state with elevated expression of CCL3 and TNF-α. These results suggest that chronic inflammatory process occurs in both male and female mouse brain during normal aging.

In this study, an in vitro experiment of human microglia exposed to CSF from the elderly or PBS was employed to explore the changes of human microglia in aged environment. Microglia extracted from an epilepsy patient showed obvious pro-inflammatory transformation in vitro. A recent study showed that young CSF restores oligodendrogenesis and memory functions in aged mice via Fgf17 [48], stressing the significance of identifying the changes of aged CSF and the mediators that promote CNS degeneration. Given the complex composition in CSF, studies with large sample size are warranted to explicit the mediators of age-related microglia response.

Although the exact link between microglia, endothelial cells, and T cell migration remains to be established, we have provided transcriptome evidence of the mechanism by which T cells infiltrate the aged brain and suggest avenues through which to maintain brain homeostasis during normal aging.

## Conclusions

**Fig. 7:**
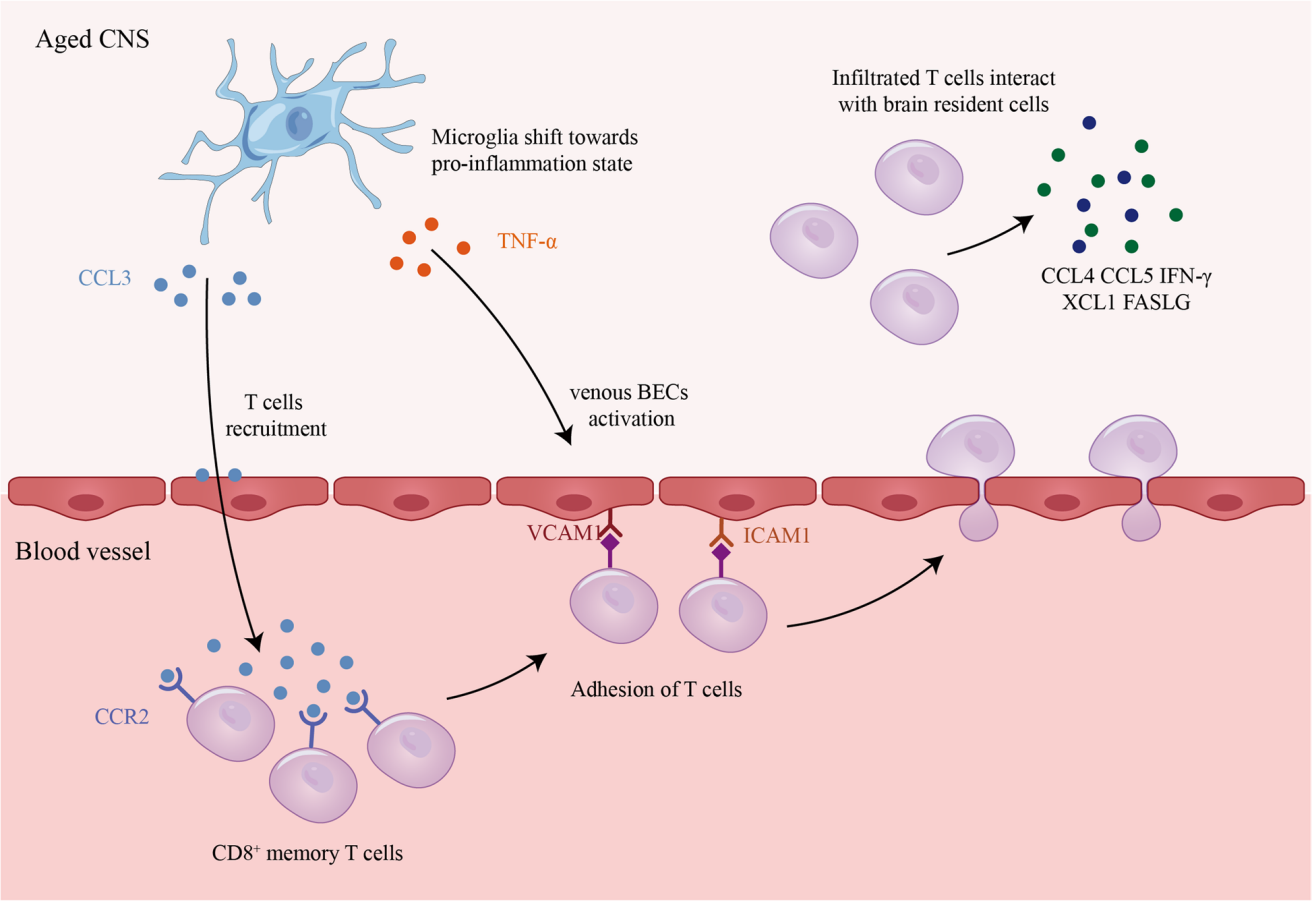
Microglia promotes T cell infiltration in the aged CNS. Microglia shift towards a pro-inflammatory state in the aged CNS and promote CD8^+^ memory T cell recruitment by releasing CCL3. Aged microglia release TNF-α, which upregulates the expression of VCAM1 and ICAM1 in venous BECs. The activated venous BECs adhere tightly to hematogenous CD8^+^ memory T cells and promote their transmigration into the CNS. The infiltrated T cells interact with brain resident cells by releasing CCL4, CCL5, IFN-γ, XCL1, FASLG

In conclusion, this study applied cell-cell interaction analysis and identified the crucial role of aged microglia in the aging process. They promote T cell infiltration by releasing chemokines and upregulating adhesion molecules on venous BECs during normal aging (Fig. 7). These findings may provide potential therapeutic targets for reducing the age-related T cell infiltration and minimizing their damage to the CNS.

## Supplementary Information

**Additional file 1: Supplementary Fig. 1.** Functional implication of infiltrated T cells. (a) Schematic illustration of original processing steps by Dulken et al. Single-cell RNA sequencing data of the SVZ from three young (3 months old) and three old (28–29 months old) mice were used for downstream analysis. (b–c) t-SNE projection of whole cells in the SVZ from young and aged mice (B). CD45^+^ cells were extracted for sub-clustering analysis (c). (d) Heatmap showing the expression levels of top five marker genes in each cell type. The most significantly upregulated genes are shown in yellow and downregulated genes are shown in Tibetan blue. (e) Feature plot depicting the expression levels of T cell markers. (f) Number of CD8^+^ T cells per coronal section in five young (6–8 weeks old) and five old (18 months old) female mice. **P = 0.0079, Mann–Whitney test, two sided. Data are shown as mean ± s.e.m. (g) Functional enrichment analysis was performed using Metascape on the T cells’ DEGs in the old SVZ. The bar plot shows the significance of the enriched terms. (h) Sankey diagram depicting the interaction between T cells and resident cells in the SVZ based on the cytokines released by T cells. The proportional flow represents the number of gene pairs. Gene pairs are listed in Supplementary Table 3. **Supplementary Fig. 2.** Single-cell transcriptomic analysis of CD8^+^ T cells. (a) Schematic illustration of original processing steps by Kimmel et al. Single-cell RNA sequencing data of the spleen from four young (7–8 months old) and three old (22–23 months old) mice were used for downstream analysis. (b) t-SNE projection of whole cells in the spleen from young and aged mice (left). CD8^+^ T cells were extracted for subclustering analysis (right). (c) Expression profiles of *Cd44* (encoding the CD44 antigen) and *Sell* (encoding the CD62L antigen) in CD8^+^ T cells are shown using the UMAP visualization approach. (d) Flow cytometry gating strategy for CD45^+^CD3^+^CD8a^+^ CD44^+^ CCR2^+^ memory T cells. (e) Protein expression of CCR2 by splenic CD8^+^ memory T cells of five young (6–8 weeks old) and five aged (18 months old) female mice. **P = 0.0054, two-tailed Student’s t test. Data are shown as mean ± s.e.m. (f) Quantification of CCL3 fluorescence in IBA1+ cells in five young (6–8 weeks old) and five aged (18 months old) female mice. ****P < 0.0001, two-tailed Student’s t test. Data are shown as mean ± s.e.m. (g) Left: Representative confocal microscopic images of young and old SVZs stained for CCL4 and IBA-1. Nuclei are labeled with DAPI. Scale bars: 200 μm or 25 μm. Right: Quantification of CCL4 fluorescence in IBA1^+^ cells in four young (6–8 weeks old) and four aged (18 months old) male mice. ***P = 0.0005, two-tailed Student’s t test. Data are shown as mean ± s.e.m. **Supplementary Fig. 3.** Single-cell transcriptomic analysis of brain endothelial cells. (a) Representative confocal microscopic images of young and old mouse brains stained for CD31 and CCL3. Nuclei are labeled with DAPI. Scale bars: 50 μm. (b) Schematic illustration of original processing steps by Dulken et al. Single-cell RNA sequencing data of the SVZ from three young (3 months old) and three old (28–29 months old) mice were used for downstream analysis. (c) t-SNE projection of whole cells in the SVZ from young and aged mice (left). Endothelial cells were extracted for subclustering analysis. (d) Plots showing the expression of representative well-known arterial (*Fbln5*), capillaries (*Rgcc*), and venous (*Nr2f2*) marker genes on the pseudotime trajectory. (e) Cellular trajectory of all endothelial cell subclusters (E0–E6) generated by Monocle. (f) Ternary diagram showing the marker genes of arteries, capillaries, and veins. Red nodes represent arterial marker genes, green nodes represent capillary marker genes, and blue nodes represent venous marker genes. (g) Expression profiles of *Cldn5* and *Ocln* in both young and aged BECs are shown using the t-SNE visualization approach. **Supplementary Fig. 4.** Single cell transcriptomic analysis of micrglia. (a) Schematic illustration of original processing steps by Dulken et al. Single-cell RNA sequence data of SVZ from three young (3 months old) and three old (28–29 months old) mice were used for downstream analysis. (b) T-SNE projection of whole cells in SVZ from young and aged mice (left). Microglia were extracted for sub-clustering analysis. (c) volcano plot depicting the DEGs in microglia cluster 2 compared to cluster 0. DEGs were colored (red for upregulated DEGs and green for downregulated DEGs). (d) Functional enrichment analysis was performed using Metascape on the upregulated DEGs in (c). The significantly overrepresented (*P* < 0.01) terms were grouped into color-coded clusters based on their membership similarities and rendered as a network plot. Each node represents an enriched term, and one representative term is shown for each cluster. Terms with a similarity > 0.3 are connected by edges. (e) The relationship between the enriched terms in (d) and the involved genes is depicted. Circos plot shows genes related to inflammatory response and cytokine production were explored for their involvement in five functional sub-categories. (f) Quantification of TNF-α fluorescence in IBA1+ cells in five young (6–8 weeks old) and five aged (18 months old) female mice. ****P < 0.0001, two-tailed Student’s t test. Data are shown as mean ± s.e.m. (g) Design foe brain slice – CD8^+^ T cells coculture experiment for flow cytometry analysis.

**Additional file 2: Supplementary Table 1.** Enriched functional terms of the infiltrated T cells.

**Additional file 3: Supplementary Table 2.** Interaction between T cells and resident cells in SVZ (R-HSA-198933 restricted).

**Additional file 4: Supplementary Table 3.** Interaction between T cells and resident cells in SVZ (cytokine restricted).

**Additional file 5: Supplementary Table 4.** Marker genes of brain endothelial cells from different vascular segments.

**Additional file 6: Supplementary Table 5.** Differentially expressed genes (CSF treated microglia vs PBS treated microglia).

## Data Availability

The datasets used and/or analyzed during the current study are available from the corresponding author on reasonable request. InterCellDB is publicly accessible as a R package in GitHub (https://github.com/ZJUDBlab/InterCellDB).

## Abbreviations

SVZ: Subventricular zone
BBB: Blood–brain barrier
CNS: Central nervous system
BECs: Brain endothelial cells
UMAP: Uniform manifold approximation and projection
t-SNE: T-distributed stochastic neighbor embedding
DEGs: Differentially expressed genes
GSEA: Gene set enrichment analysis
CSF: Cerebrospinal fluid
BMECs: Brain microvascular endothelial cells
