# Supplementary material for: Aged microglia promote peripheral T cell infiltration by reprogramming the microenvironment of neurogenic niches
Source: Immun Ageing. 2022 Jul 25;19:34. doi: 10.1186/s12979-022-00289-6 (PMC9310471; doi:10.1186/s12979-022-00289-6)
Supplement: Supplementary file 1 — Additional file 1: Supplementary Fig. 1. Functional implication of infiltrated T cells. (a) Schematic illustration of original processing steps by Dulken et al. Single-cell RNA sequencing data of the SVZ from three young (3 months old) and three old (28–29 months old) mice were used for downstream analysis. (b–c) t-SNE projection of whole cells in the SVZ from young and aged mice (B). CD45+ cells were extracted for sub-clustering analysis (c). (d) Heatmap showing the expression levels of top five marker genes in each cell type. The most significantly upregulated genes are shown in yellow and downregulated genes are shown in Tibetan blue. (e) Feature plot depicting the expression levels of T cell markers. (f) Number of CD8+ T cells per coronal section in five young (6–8 weeks old) and five old (18 months old) female mice. **P = 0.0079, Mann–Whitney test, two sided. Data are shown as mean ± s.e.m. (g) Functional enrichment analysis was performed using Metascape on the T cells’ DEGs in the old SVZ. The bar plot shows the significance of the enriched terms. (h) Sankey diagram depicting the interaction between T cells and resident cells in the SVZ based on the cytokines released by T cells. The proportional flow represents the number of gene pairs. Gene pairs are listed in Supplementary Table 3. Supplementary Fig. 2. Single-cell transcriptomic analysis of CD8+ T cells. (a) Schematic illustration of original processing steps by Kimmel et al. Single-cell RNA sequencing data of the spleen from four young (7–8 months old) and three old (22–23 months old) mice were used for downstream analysis. (b) t-SNE projection of whole cells in the spleen from young and aged mice (left). CD8+ T cells were extracted for subclustering analysis (right). (c) Expression profiles of Cd44 (encoding the CD44 antigen) and Sell (encoding the CD62L antigen) in CD8+ T cells are shown using the UMAP visualization approach. (d) Flow cytometry gating strategy for CD45+CD3+CD8a+ CD44+ CCR2+ memory T cel [file 12979_2022_289_MOESM1_ESM.docx]

**Supplementary Information for**

**Aged Microglia Promote Peripheral T Cell Infiltration by Reprogramming the Microenvironment of Neurogenic Niches**

Xiaotao Zhang, Rui Wang, Haoran Chen, Chenghao Jin, Ziyang Jin, Jianan Lu, Liang Xu, Yunrong Lu, Jianmin Zhang, and Ligen Shi

^#^ To whom correspondence should be addressed:

Dr. Ligen Shi, Department of Neurosurgery, Second Affiliated Hospital, School of Medicine, Zhejiang University, 88 Jiefang Road, Hangzhou, Zhejiang 310009, China. Tel: +86-571-87784715; Fax: +86-571-87784755; email: [slg0904@zju.edu.cn](mailto:slg0904@zju.edu.cn).

Dr. Jianmin Zhang, Department of Neurosurgery, Second Affiliated Hospital, School of Medicine, Zhejiang University, 88 Jiefang Road, Hangzhou, Zhejiang 310009, China. Tel: +86-571-87784715; Fax: +86-571-87784755; email: [zjm135@zju.edu.cn](mailto:zjm135@zju.edu.cn).

**This file includes:**

**Supplementary Figures 1-4**

**Supplementary Tables 1-6**

**
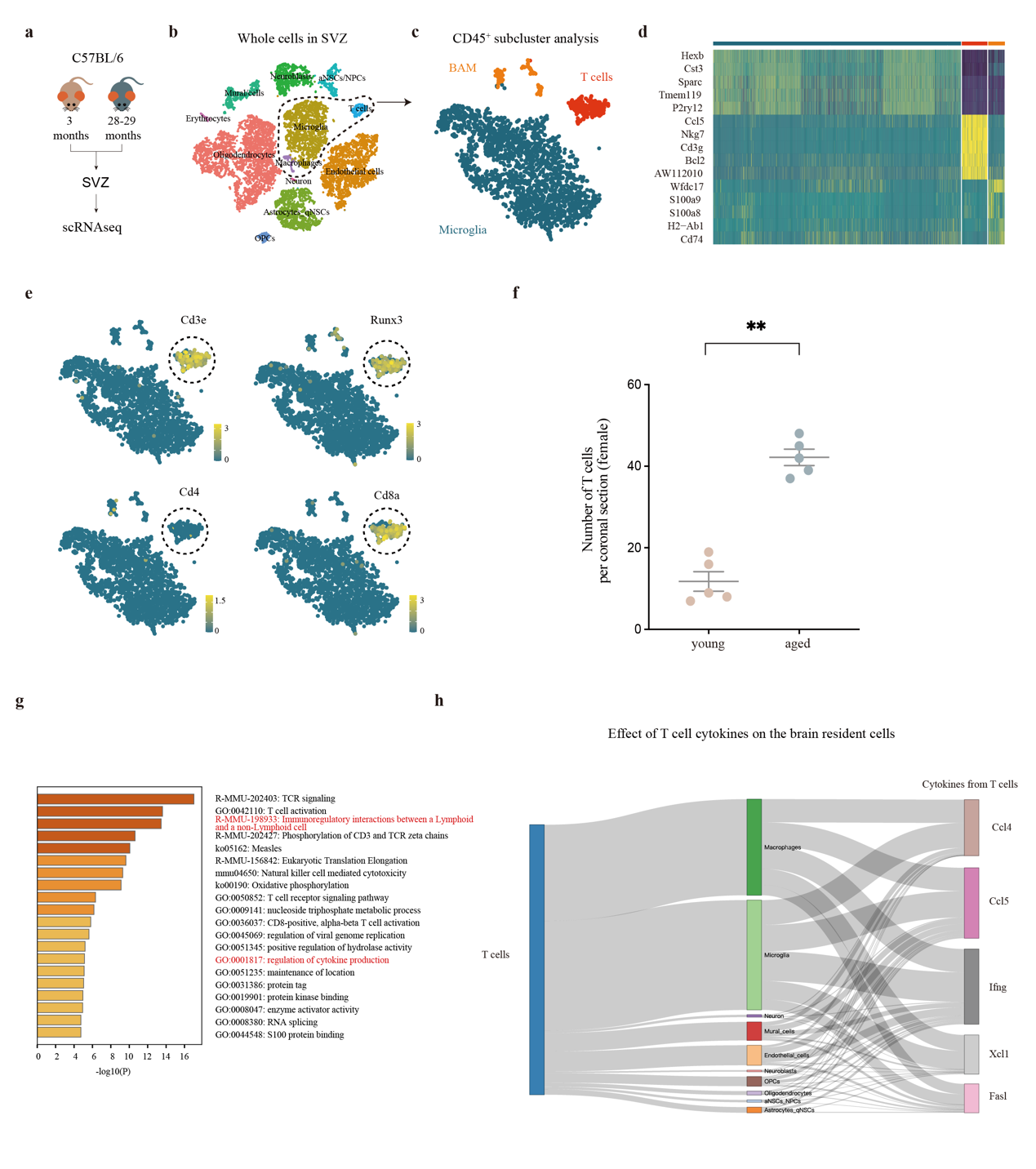
**

**Supplementary Figure 1 | Functional implication of infiltrated T cells. (a)** Schematic illustration of original processing steps by Dulken et al. Single-cell RNA sequencing data of the SVZ from three young (3 months old) and three old (28–29 months old) mice were used for downstream analysis. **(b–c)** t-SNE projection of whole cells in the SVZ from young and aged mice (B). CD45^+^ cells were extracted for sub-clustering analysis (c). **(d)** Heatmap showing the expression levels of top five marker genes in each cell type. The most significantly upregulated genes are shown in yellow and downregulated genes are shown in Tibetan blue. **(e)** Feature plot depicting the expression levels of T cell markers. **(f)** Number of CD8^+^ T cells per coronal section in five young (6–8 weeks old) and five old (18 months old) female mice. **P = 0.0079 , Mann–Whitney test, two sided. Data are shown as mean ± s.e.m. **(g)**Functional enrichment analysis was performed using Metascape on the T cells’ DEGs in the old SVZ. The bar plot shows the significance of the enriched terms. **(h)** Sankey diagram depicting the interaction between T cells and resident cells in the SVZ based on the cytokines released by T cells. The proportional flow represents the number of gene pairs. Gene pairs are listed in Supplementary Table 3.


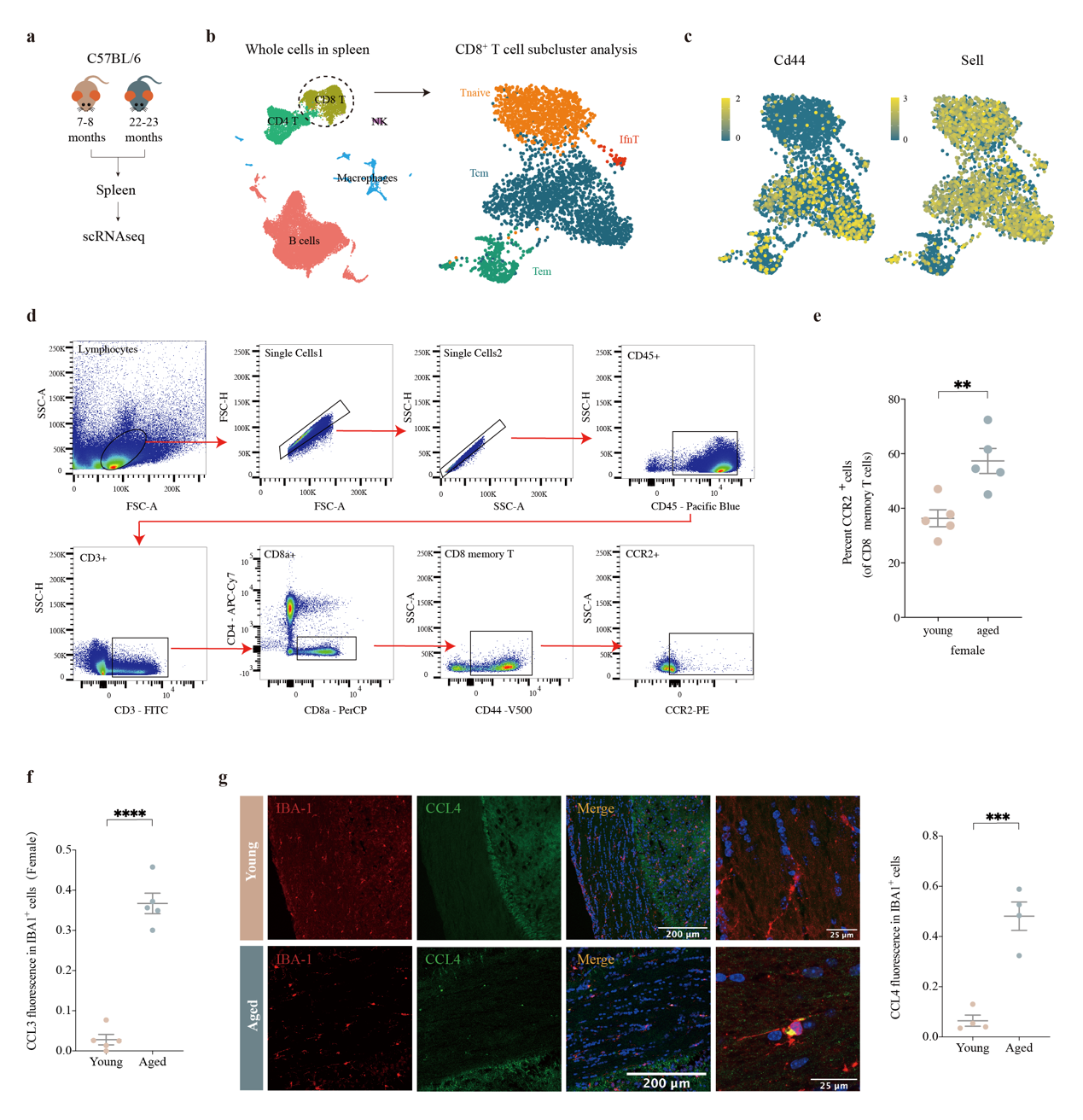


**Supplementary Figure 2 | Single-cell transcriptomic analysis of CD8^+^ T cells. (a)** Schematic illustration of original processing steps by Kimmel et al. Single-cell RNA sequencing data of the spleen from four young (7–8 months old) and three old (22–23 months old) mice were used for downstream analysis. **(b)** t-SNE projection of whole cells in the spleen from young and aged mice (left). CD8^+^ T cells were extracted for subclustering analysis (right). **(c)** Expression profiles of *Cd44* (encoding the CD44 antigen) and *Sell* (encoding the CD62L antigen) in CD8^+^ T cells are shown using the UMAP visualization approach. **(d)** Flow cytometry gating strategy for CD45^+^CD3^+^CD8a^+^ CD44^+^ CCR2^+^ memory T cells. **(e)** Protein expression of CCR2 by splenic CD8^+^ memory T cells of five young (6–8 weeks old) and five aged (18 months old) female mice. **P = 0.0054 , two-tailed Student’s t test. Data are shown as mean ± s.e.m. **(f)** Quantification of CCL3 fluorescence in IBA1+ cells in five young (6–8 weeks old) and five aged (18 months old) female mice. ****P < 0.0001, two-tailed Student’s t test. Data are shown as mean ± s.e.m. **(g)** Left: Representative confocal microscopic images of young and old SVZs stained for CCL4 and IBA-1. Nuclei are labeled with DAPI. Scale bars: 200 μm or 25 μm. Right: Quantification of CCL4 fluorescence in IBA1^+^ cells in four young (6–8 weeks old) and four aged (18 months old) male mice. ***P = 0.0005, two-tailed Student’s t test. Data are shown as mean ± s.e.m.


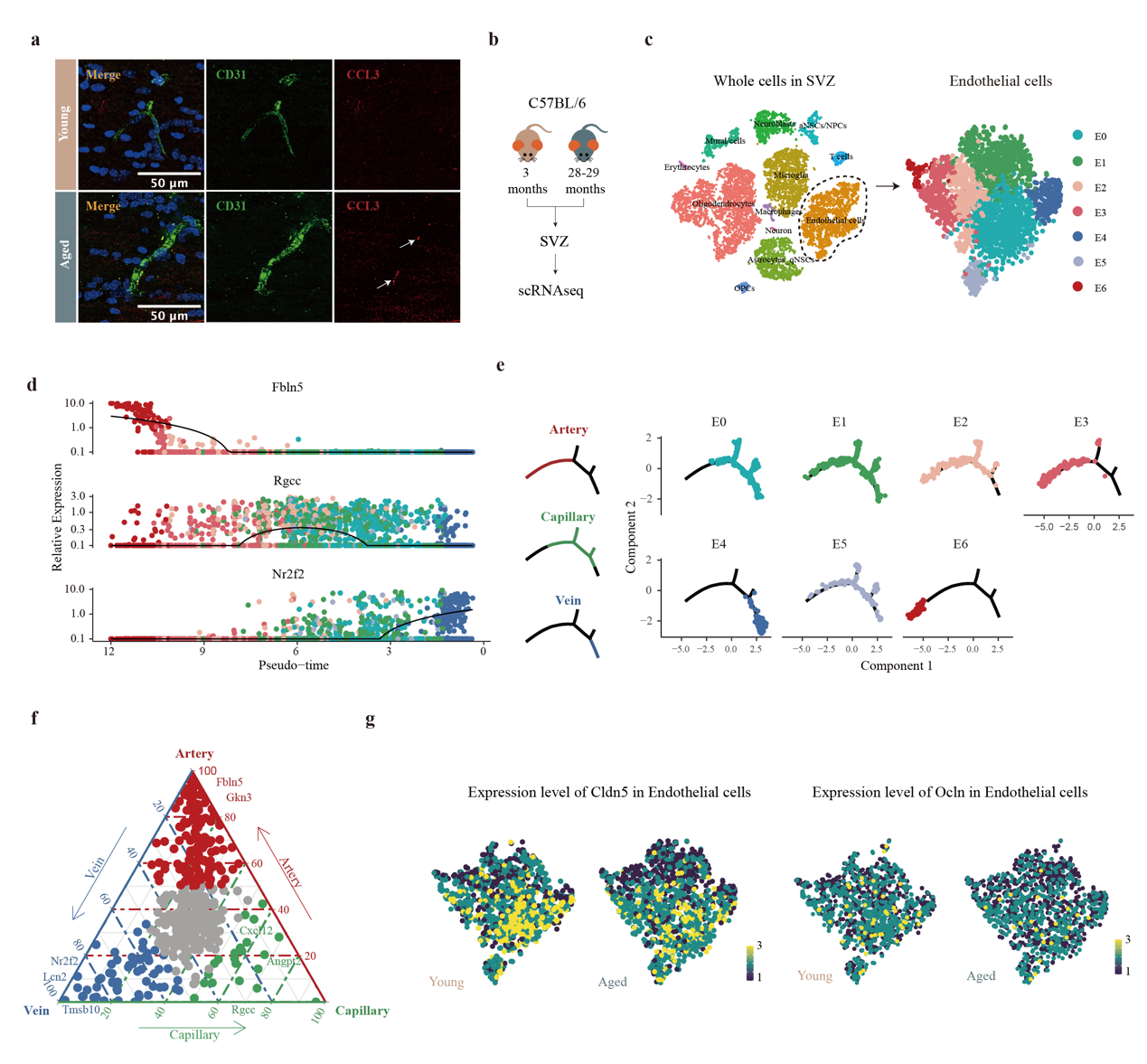


**Supplementary Figure 3 | Single-cell transcriptomic analysis of brain endothelial cells. (a)** Representative confocal microscopic images of young and old mouse brains stained for CD31 and CCL3. Nuclei are labeled with DAPI. Scale bars: 50 μm. **(b)** Schematic illustration of original processing steps by Dulken et al. Single-cell RNA sequencing data of the SVZ from three young (3 months old) and three old (28–29 months old) mice were used for downstream analysis. **(c)** t-SNE projection of whole cells in the SVZ from young and aged mice (left). Endothelial cells were extracted for subclustering analysis. **(d)** Plots showing the expression of representative well-known arterial (*Fbln5*), capillaries (*Rgcc*), and venous (*Nr2f2*) marker genes on the pseudotime trajectory. **(e)** Cellular trajectory of all endothelial cell subclusters (E0–E6) generated by Monocle. **(f)** Ternary diagram showing the marker genes of arteries, capillaries, and veins. Red nodes represent arterial marker genes, green nodes represent capillary marker genes, and blue nodes represent venous marker genes. **(g)** Expression profiles of *Cldn5* and *Ocln* in both young and aged BECs are shown using the t-SNE visualization approach.


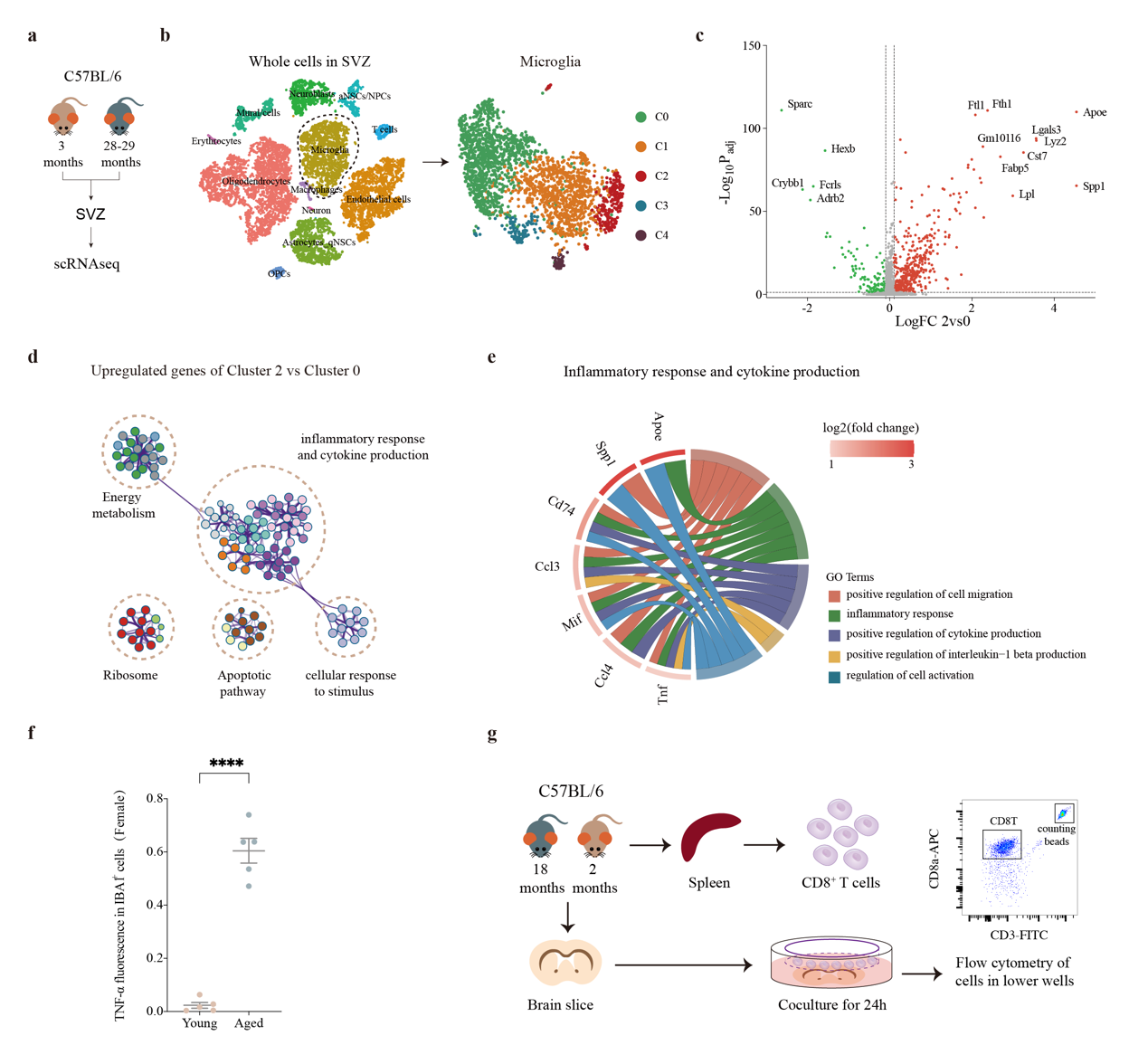


**Supplementary Figure 4 | Single cell transcriptomic analysis of micrglia. (a)** Schematic illustration of original processing steps by Dulken et al. Single-cell RNA sequence data of SVZ from three young (3 months old) and three old (28-29 months old) mice were used for downstream analysis. **(b)** T-SNE projection of whole cells in SVZ from young and aged mice (left). Microglia were extracted for sub-clustering analysis. **(c)** volcano plot depicting the DEGs in microglia cluster 2 compared to cluster 0. DEGs were colored (red for upregulated DEGs and green for downregulated DEGs). **(d)** Functional enrichment analysis was performed using Metascape on the upregulated DEGs in (c). The significantly overrepresented (P<0.01) terms were grouped into color-coded clusters based on their membership similarities and rendered as a network plot. Each node represents an enriched term, and one representative term is shown for each cluster. Terms with a similarity > 0.3 are connected by edges. **(e)** The relationship between the enriched terms in (d) and the involved genes is depicted. Circos plot shows genes related to inflammatory response and cytokine production were explored for their involvement in five functional sub-categories. **(f)** Quantification of TNF-α fluorescence in IBA1+ cells in five young (6–8 weeks old) and five aged (18 months old) female mice. ****P < 0.0001, two-tailed Student’s t test. Data are shown as mean ± s.e.m. **(g)** Design foe brain slice – CD8^+^ T cells coculture experiment for flow cytometry analysis.

**Supplementary Tables 1-6**

**Supplementary Table 1 | Enriched functional terms of the infiltrated T cells.**

Please see attachment.

**Supplementary Table 2 | Interaction between T cells and resident cells in SVZ (R-HSA-198933 restricted).**

Please see attachment.

**Supplementary Table 3 | Interaction between T cells and resident cells in SVZ (cytokine restricted).**

Please see attachment.

**Supplementary Table 4 | Marker genes of brain endothelial cells from different vascular segments.**

Please see attachment.

**Supplementary Table 5 | Differentially expressed genes (CSF treated microglia vs PBS treated microglia)**

Please see attachment.

**Supplementary Table 6 | Statistics reporting.**

|  | | | | |
| --- | --- | --- | --- | --- |
| **Fig NO.** | **Description** | **Group (n)** | **Test used** | **Degree of freedom and P value** |
| Fig 1b | T cells proportion | Young(3) aged(3) | one-tailed Wilcoxon rank sum test) | *p=0.05 |
| Fig 1d | Number of CD8^+^ T cells per coronal section (male) | young(4) aged(4) | Mann–Whitney test, two sided | *P = 0.0286 |
| Supplementary Fig 1f | Number of CD8^+^ T cells per coronal section (female) | young(5) aged(5) | Mann–Whitney test, two sided | **P = 0.0079 |
| Fig 2b | expression level of  Cxcr3 Ccr2 Ccr5 | CD4^+^T: young(1836) aged(2472) CD8^+^T: young(1817) aged(1501) | Wilcoxon rank sum test,  Bonferroni correction | ***p<0.001 |
| Fig 2d | Protein expression of CCR2 by splenic CD8+ memory T cells(male) | young(4) aged(4) | two-tailed Student’s t test | *P = 0.0264 |
| Fig 2e | expression level of  Ccl3 Ccl4 | microglia:young(1218) aged(1198) | Wilcoxon rank sum test,  Bonferroni correction | Ccl3: ***adjusted p=6.55×10^-9^ |
| Fig 2f | human CCL3 plasma level | 4263 young adults to nonagenarians | F-test,  Benjamini–Hochberg | ***q-value = 1.14×10^-31^ |
| Fig 2g | Quantification of CCL3 fluorescence in IBA1+ cells(male) | young(4) aged(4) | two-tailed Student’s t test | ****P < 0.0001 |
| Supplementary Fig 2e | Protein expression of CCR2 by splenic CD8+ memory T cells(female) | young(5) aged(5) | two-tailed Student’s t test | *P = 0.0054 |
| Supplementary Fig 2f | Quantification of CCL3 fluorescence in IBA1+ cells(female) | young(5) aged(5) | two-tailed Student’s t test | ****P < 0.0001 |
| Supplementary Fig 2g | Quantification of CCL4 fluorescence in IBA1+ cells(male) | young(4) aged(4) | two-tailed Student’s t test | ***P = 0.0005 |
| Fig 3b | expression level of Ocln Cldn5 | capillary:young(756) aged(709) | Wilcoxon rank sum test,  Bonferroni correction | Ocln: **adjusted p=0.0038 Cldn5: ***adjusted p=1.52×10^-7^ |
| Fig 3d | Quantitative analysis of IgG extravascular | young(4) aged(4) | two-tailed Student’s t test | *P = 0.0117 |
| Fig 4g | quantification of VCAM1 colocalization with CD31 | young(4) aged(4) | two-tailed Student’s t test | **P = 0.0096 |
| Fig 4h | quantification of ICAM1 colocalization with CD31 | young(4) aged(4) | two-tailed Student’s t test | ***P = 0.0008 |
| Fig 5f | Quantification of TNF-α fluorescence in IBA1+ cells(male) | young(4) aged(4) | two-tailed Student’s t test | ***P = 0.0008 |
| Supplementary Fig 5f | Quantification of TNF-α fluorescence in IBA1+ cells(female) | young(5) aged(5) | two-tailed Student’s t test | ****P < 0.0001 |
| Fig 5g | CD8 T cells migration number/ count beads | n=5 | One-way ANOVA | *Adjusted-P = 0.0155  #Adjusted-P = 0.0161 |
